# Approaches used to model patient and carer health-related quality of life in economic models of rare disease treatments in NICE appraisals

**DOI:** 10.1186/s12955-026-02532-w

**Published:** 2026-04-13

**Authors:** Lea Wiedmann, John Cairns

**Affiliations:** Department of Health Services Research and Policy, London School of Hygiene & Tropical Medicine, London, UK

**Keywords:** NICE, England, Rare diseases, Health-related quality of life, Health technology assessment, EQ-5D

## Abstract

**Introduction:**

Providing robust health-related quality of life (HRQoL) evidence for rare disease treatments (RDTs) is difficult due to the challenges involved in generating and interpreting evidence for rare diseases. Our objective was to systematically review the approaches used to model patient and carer HRQoL in economic models in RDT appraisals published by the National Institute for Health and Care Excellence (NICE).

**Methods:**

We identified appraisals completed between 2011 and 2023 and recorded HRQoL modelling characteristics from appraisal documents, including the approach used to derive the patient health state utility values (HSUVs) used in the final appraisal, and whether and how carer HRQoL was included. We mapped the approaches to estimating patient and carer HRQoL against NICE’s hierarchy of preferred HRQoL methods.

**Results:**

We identified a total of 111 indications. Overall, we found heterogeneity in the approaches used to model patient and carer HRQoL. Regarding patient HRQoL, EQ-5D data was not available from a relevant study in 59.5% of indications in the final appraisal, and EQ-5D was not deemed appropriate by the committee in 6.3% of indications. EQ-5D from a relevant study was available and used in 34.2% of indications. Various sources of patient utilities were used, including EQ-5D values from a relevant study, estimates from the published literature (EQ-5D or non-EQ-5D), values derived from statistical mapping, vignette studies, or proxy conditions, combinations of several types, or values based on assumptions. Approaches to modelling patient utilities were also discussed in three-quarters of final appraisal documents, suggesting a considerable influence on the final recommendation. The impact on carer HRQoL was included quantitatively in the final appraisal in 26.1% of indications; for 3.6% of indications, the committee considered it qualitatively; and for one indication (0.9%) both quantitatively and qualitatively.

**Conclusion:**

Our findings suggest that, given the NICE hierarchy, patient and carer HRQoL was not very well captured in RDT appraisals. If the NICE hierarchy is viewed as a taxonomy of possible approaches, our findings also suggest that it does not accommodate some of the approaches used to estimate HRQoL in RDT appraisals. These issues raise questions about the consistency with which HRQoL is modelled and reflect the challenges of generating robust HRQoL evidence for RDTs.

**Supplementary Information:**

The online version contains supplementary material available at 10.1186/s12955-026-02532-w.

## Introduction

Most rare diseases are genetic, chronic and progressive in nature, have heterogenous disease characteristics and lack effective treatments [1, 2]. They often lead to severe impairment in physical, communication, and cognitive functions, affecting the health-related quality of life (HRQoL) of those affected and their carers [3]. HRQoL of rare disease patients is poorer than for those living with prevalent chronic conditions [4, 5]. This may be explained by diagnostic delays, the lack of specialised clinical knowledge, limited treatment options and pathways, and insufficient resources and facilities for conducting research and providing psychosocial support [4, 5]. In addition, patients with lower prevalence rare conditions have poorer HRQoL outcomes compared to patients with more common rare conditions [6], highlighting the HRQoL burden for patients with very rare diseases. As many rare diseases have exclusively paediatric onset (approximately 70%) [1], family members also often act as informal carers, which can cause a substantial burden [7, 8]. HRQoL of carers is significantly influenced by patient age and HRQoL, as well as by their self-perceived carer burden [9].

HRQoL is typically included in cost-effectiveness analyses used by health technology assessment bodies, such as the National Institute for Health and Care Excellence (NICE) in England, to inform their decisions about allocating resources within the healthcare system. This includes decisions about adopting or rejecting specific health technologies. The scarcity of HRQoL data for RDTs has been identified as a particular challenge for economic models of RDTs, leading to high levels of uncertainty in reimbursement decisions [10]. Measurement of HRQoL in rare diseases and of the potential HRQoL benefit of RDTs is often difficult because of difficulty measuring HRQoL in paediatric populations, small sample sizes, and unique or heterogenous symptoms [11].

HRQoL is usually included in the form of health state utility values (HSUVs) in economic models (henceforth referred to as ‘utilities’) and can be derived from generic preference-based instruments such as the EuroQoL-5 Dimension (EQ-5D) tool [12]. Generic, preference-based HRQoL instruments are standardised tools to measure, compare and value HRQoL across different conditions and populations [13]. Such instruments specify generic health states for which utilities are estimated using a preference-based scoring algorithm. However, there are challenges associated with using generic preference-based instruments such as the EQ-5D in rare diseases [14–16], including the lack of sensitivity of generic instruments in capturing key symptoms and health consequences of rare conditions, resulting in the exclusion of important aspects relevant to those affected [17, 18]. Alternative options include deriving utilities from condition-specific preference-based instruments, mapping of results of condition-specific instruments to the EQ-5D, or identifying suitable data from the published literature, vignette studies, or ‘proxy’ conditions [15, 19, 20].

In this context, several studies have reviewed approaches used to include carer HRQoL in NICE appraisals [21, 22], or patient or carer HRQoL in selected NICE appraisals for RDTs [23–26]. However, a comprehensive analysis of modelling approaches of patient and carer HRQoL used in economic models for RDTs specifically is lacking. There is also a lack of comparison between the modelling of HRQoL in cost-effectiveness analyses of RDTs in appraisals and NICE’s hierarchy of preferred HRQoL methods [27]. Therefore, this study sought to close this evidence gap by systematically reviewing the approaches used for modelling patient and carer HRQoL in economic models in appraisals of RDTs. To do so, we review the utilities used in economic models for RDT appraisals that have been published under NICE’s Technology Appraisal (TA) and Highly Specialised Technology (HST) guidance between 2011 and 2023. We then compare our findings with NICE’s published hierarchy of preferred HRQoL methods and discuss how HRQoL modelling practices in RDT appraisals differ from this hierarchy.

We provide a list of abbreviations in S1.

## Methods

### NICE’s hierarchy of preferred HRQoL methods

NICE recommends that health effects in cost-utility analyses should be expressed in quality-adjusted life years (QALYs) and that HRQoL should be reported directly by patients or their carers [27]. In addition, the collected data should be valued based on a valuation of preferences from a representative sample of the UK general public obtained using a choice-based method [27].

The NICE reference case also states that the EQ-5D is the preferred generic instrument to estimate utilities [27]. If EQ-5D is not available from relevant clinical trials, utilities should be derived either from the published literature or from statistical mapping. The latter can be an option when utilities are only available from a condition-specific instrument, for example, but the health technology assessment body requires the use of a generic tool [28]. Thus, mapping involves estimating the relationship between a source and a target measure, typically using a regression equation [29].

**Fig. 1:**
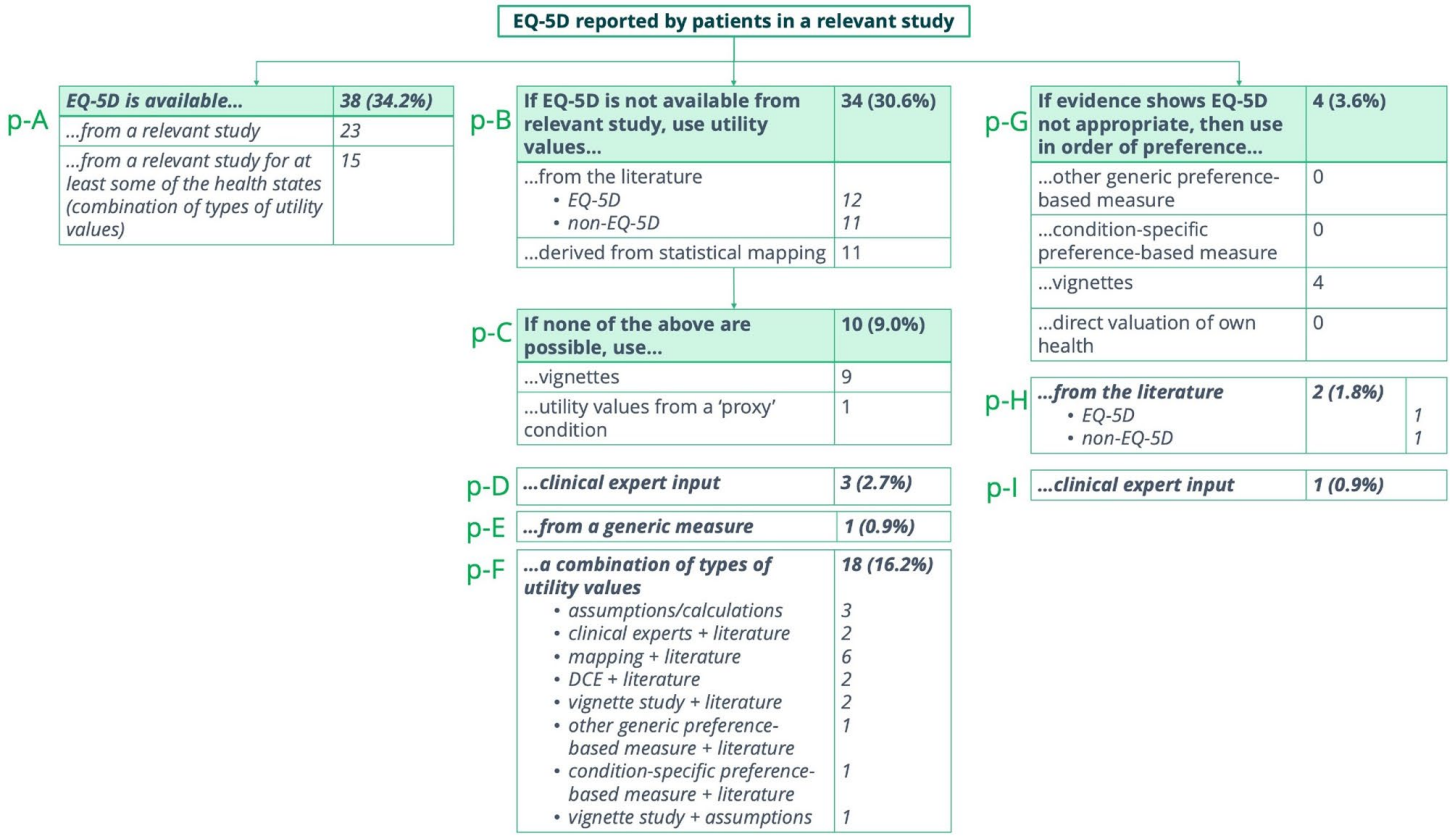
Types of patient utilities (*n* = 111) notes: categories are labelled p-A to p-I, where “p” denotes patient utilities and letters (A–I) are used for distinction. Categories shown in italics were added by the authors. Categories p-D, p-E, p-F, p-H, and p-I fall outside the NICE hierarchy

Alternatively, if it is not possible to derive EQ-5D from a literature source or through mapping, NICE will consider vignettes or the use of utilities from a proxy condition [27]. Vignettes are hypothetical descriptions of health states that are valued using a preference elicitation technique to obtain utility values [30]. Various techniques can be employed to value vignettes, with the most common ones being the Time Trade-Off method or the standard gamble [30]. The valuation is usually completed by non-patient populations [19], such as members of the general public or clinical experts. NICE recommends that the EQ-5D should be completed based on the vignette by members of the public or people with the condition and valued using the EQ-5D value set [27]. If the evidence shows that the EQ-5D is not appropriate, NICE recommends using other generic or condition-specific preference-based measures [27]. This is followed by vignettes valued by members of the general public using an appropriate elicitation technique, such as the Time Trade-Off, or a direct valuation of own health by patients (see S2, Fig. 1 for a schematic overview of NICE’s hierarchy of preferred HRQoL methods) [27].

Furthermore, the NICE guidance manual does not specify a requirement to include health effects of carers in economic modelling evidence, but evaluations will consider this impact when relevant [27]. If included, NICE advises manufacturers to show the effect of the condition on carer HRQoL and the impact of the technology on carers in its submission [27], but further guidance or best practice examples are lacking.

### Appraisal selection

We identified RDT appraisals completed between 2011 and 2023 using the Orphan Register of the Medicines & Healthcare products Regulatory Agency (MHRA) [31] because the NICE guidance database does not enable filtering for RDTs. If an appraisal published under the TA guidance process was available for a health technology listed in the Orphan Register, it was considered an RDT appraisal. In addition, we considered all HST appraisals for inclusion. Appraisals for RDTs with an expired market exclusivity period were included if the appraisal was published before the exclusivity period had ended. Terminated appraisals, appraisals in which the manufacturer withdrew the submission, multiple technology appraisals, appraisals which had been replaced by updated guidance, and cost comparisons were excluded. Further, two indications (nusinersen and risdiplam for pre-symptomatic spinal muscular atrophy) were excluded because the manufacturer did not submit cost-effectiveness evidence for these indications (see S3 for additional detail on the appraisal selection process and S4 for a list of included appraisals).

### Approach to data extraction and categorisation

NICE appraisal documents were the primary data source for the analysis. Documents included the final scope, NICE committee papers which include the manufacturer’s submission, the final appraisal determination (FAD) or final evaluation determination (FED), and public committee presentations.

For each RDT indication we recorded appraisal characteristics. These included the recommendation by the committee specified in the FAD or FED. Possible recommendations include a recommendation in line with the marketing authorisation or in line with use in clinical practise in the National Health Service (NHS) (‘recommended’), recommendations in which the eligible population was further restricted to a subset of the licensed population (‘optimised’), negative recommendations (‘not recommended’), or recommendations for research purposes only (‘recommended only in research’). In addition, health technologies targeting oncological conditions can be recommended in the Cancer Drugs Fund [32] and health technologies targeting non-oncological conditions can be recommended under a managed access agreement while additional data are collected to address uncertainties in the evidence base. We also recorded the therapeutic area of each indication as per the NICE database [33], drawing a distinction between oncological and non-oncological conditions. An overview of the therapeutic area of all RDTs included in this analysis is provided in S5. Further, we recorded whether the RDT was appraised under the TA or the HST guidance process.

We also recorded several modelling characteristics for each indication. Regarding patient utilities, we recorded the approach used to generate patient utilities for each indication in the final appraisal. Regarding carer HRQoL, we recorded whether it was included quantitatively in the final appraisal, including the approach and sources of utility decrements (if applicable), or whether it was considered qualitatively by the committee.

It is important to note that parameters in the economic model originally submitted by the manufacturer may differ from the revised model after the technical engagement of the manufacturer with NICE, the assessment of the Evidence Assessment Group (EAG), or committee meetings. In this study, we chose to focus on the approaches taken in the final appraisal. This enabled an analysis of the model relevant for the reimbursement recommendation of the committee, while also simplifying data extraction.

### Data analysis

Our overall analysis approach was descriptive without the application of inferential analysis methods. We first provided descriptive statistics for all extracted characteristics. Then we mapped extracted data on patient and carer HRQoL against NICE’s hierarchy of preferred HRQoL methods.

We grouped indications for which EQ-5D values were available from a relevant study into category p-A (category c-A for carers). We grouped indications for which EQ-5D values were not available from a relevant study into categories p-B to p-F based on the type of approach used to generate utilities (categories c-B to c-E for carers). We defined a relevant study as a study upon which the estimate of treatment effect is based. As the NICE methods manual [27] does not provide a definition of proxy conditions, we categorised indications as being informed by proxy conditions where utilities for all health states were derived from a proxy population and this was explicitly described as such in the committee papers.

We categorise indications in which the manufacturer stated that EQ-5D data was inappropriate, and the committee did not object, into groups p-G to p-I (category c-F for carers). However, we did not consider indications in which the manufacturer argued that EQ-5D was not appropriate, but this was dismissed by the committee, such as in the appraisal of bulevirtide for chronic hepatitis D [TA896] [34], for these groups.

We chose to categorise indications based on the approach used to generate the patient utilities that were used to inform the model health states. We did not use the approach used to generate any additional (dis-)utilities, such as those associated with the treatment administration or the inclusion of adverse events, for the categorisation.

## Results

### Summary of the dataset

We identified a total of 101 appraisals (80 active substances, 111 indications). This included 75 appraisals (55 active substances, 82 indications) from the TA guidance and 26 appraisals (25 active substances, 29 indications) from the HST guidance. In the majority of appraised indications, the RDT was recommended, or an optimised recommendation was issued (Table 1). Only in very few indications, did NICE not recommend the RDT (8.1%). Overall, 50.5% of indications were for RDTs treating oncological conditions, and 26.1% of indications were appraised under the HST guidance process.

**Table 1:** Characteristics of analysed indications (*n* = 111)

	Number of indications (%)
**NICE recommendation**	
Recommended	51 (45.9)
Recommended (Cancer Drugs Fund)	6 (5.4)
Recommended (Managed Access Agreement)	1 (0.9)
Optimised	37 (33.3)
Optimised (Cancer Drugs Fund)	1 (0.9)
Optimised (Managed Access Agreement)	5 (4.5)
Recommended only in research	1 (0.9)
Not recommended	9 (8.1)
**Therapeutic area**	
Oncological	55 (49.5)
Non-oncological	56 (50.5)
**Process**	
Technology Appraisal guidance	82 (73.9)
Highly Specialised Technology appraisal guidance	29 (26.1)

NICE = National Institute for Health and Care Excellence

### Types of patient utilities

A range of approaches were used to model patient HRQoL. Overall, for 59.5% (*n* = 66) of the indications analysed, EQ-5D data was not available from a relevant study (categories p-B to p-F) (Fig. 1). In 34.2% (*n* = 38) of indications, EQ-5D was available and used (category p-A). In 6.3% (*n* = 7) of indications (p-G to p-I), the committee agreed that EQ-5D was not appropriate. Categories p-D, p-E, p-F, p-H and p-I fall outside the NICE hierarchy.

### Where EQ-5D is available from a relevant study

EQ-5D was taken from a relevant study for either all or at least some of the health states in 34.2% of the indications (see S6, Table 4 for additional detail). For example, in the appraisal of avacopan for polyangitis [TA825] all utilities were derived from the EQ-5D collected in the ADVOCATE trial except for patients with end-stage renal disease which was obtained from the published literature and estimated according to the type of treatment those patients receive [35].

### Where EQ-5D is not available from a relevant study

Where EQ-5D was not available from a relevant study, utilities were sourced from the published literature or derived from statistical mapping. However, in only about half of the indications where utilities were derived from the literature, did the literature source provide EQ-5D values (see S6, Table 5 for further detail). Statistical mapping included mapping of both generic and condition-specific instruments to the EQ-5D. For example, results from the cancer-specific EORTC QLQ-C30 instrument or values from the generic PedsQL instrument specifically developed to measure HRQoL in children and adolescents, were mapped to the EQ-5D. Moreover, if the 5 L version of the EQ-5D was used to collect HRQoL data in the trial, estimates were typically mapped to the 3 L version of the EQ-5D to comply with the NICE reference case [27].

Additionally, the manufacturer sometimes argued that there was no validated mapping function to convert HRQoL estimates collected during the trial into EQ-5D values or that this was not needed. For example, in the appraisal of obinutuzumab for untreated chronic lymphocytic leukaemia [TA343], the manufacturer argued that no validated mapping function existed for the cancer-specific EORTC QLQ-C30 instrument [36] but the EAG disagreed [37]. Similarly, in the appraisal of selumetinib for neurofibromatosis [HST20] the manufacturer argued that improvements in HRQoL were confirmed by the PedsQL scores without a need for mapped values [38] but the EAG again disagreed [39]. In both cases, the committee (partly) accepted values from vignette studies, suggesting that mapping to a preference-based measure is not always preferred.

In those cases where manufacturers used vignettes to derive utilities, they were evaluated either by members of the public, patients and/or carers, or clinical experts using the Time Trade-Off method, visual analogue scale, or the EQ-5D. Moreover, we identified discrepancies in how and by whom vignettes should be evaluated between the approaches taken in the final appraisals of RDTs and the NICE hierarchy. Rather than members of the general public or patients using the EQ-5D to value vignettes, our analysis showed that vignettes were typically evaluated by clinical experts using the EQ-5D, by members of the public using the Time Trade-Off method, or by patients and/or their carers using a visual analogue scale. In addition, when EQ-5D values are unavailable, scenario analyses using alternative sets of utility values are often conducted. For example, in the appraisal of eladocagene exuparvovec for aromatic L-amino acid decarboxylase (AADC) deficiency [HST26], the manufacturer undertook a vignette study with members of the UK general population using the Time Trade-Off method [40]. As part of its assessment, the EAG explored scenarios using two alternative sources of utility values, including those from a previous NICE appraisal for spinal muscular atrophy [HST15]. Although most of the individual utility values from the manufacturer’s vignette study were higher than those used in the earlier appraisal, applying the HST15 values resulted in a more favourable ICER for eladocagene exuparvovec. Ultimately, the committee concluded that the vignette-based values proposed by the manufacturer in HST26 represented conservative estimates and were appropriate for decision making, while noting some residual uncertainty regarding how well the vignettes captured the full impact of the condition [41].

Furthermore, with regard to proxy conditions, the manufacturer used a confidential proxy condition to inform every utility only in the appraisal of afamelanotide for erythropoietic protoporphyria [HST27] [42]. This choice followed the committee’s rejection of data from a condition-specific quality of life questionnaire, the EPP-QoL, developed by the manufacturer [42]. In other appraisals, the utilities were derived from proxy conditions for some health states. For example, in the appraisal of givosiran for acute hepatic porphyria [HST16], the committee agreed with the EAG that the most appropriate utilities for modelling chronic symptoms of the condition can be taken from relapsing-remitting multiple sclerosis as a proxy condition [43]. Another example is the appraisal of Holoclar treating limbal stem cell deficiency after eye burns [TA467] in which a utility for cataracts was used as a proxy for disfigurement [44]. Appraisals in which some utilities were derived from proxy conditions are highlighted in S6.

### Where EQ-5D is not appropriate

In appraisals in which the committee agreed that the EQ-5D was not appropriate, the manufacturer derived utilities from vignettes, the published literature, or clinical experts (see S6, Table 6 for further detail). In these cases, EQ-5D data were available for the initial submission but were eventually not used in the final economic model because of an absence of values for some health states, no significant changes from baseline or between trial arms in the EQ-5D, or because they were not deemed clinically plausible.

### Combination of types of utilities

When evidence for specific health states or patient subgroups was limited, manufacturers typically combined different types (see S6 for further detail). In 29.7% (33/111) of indications, utilities were derived from a combination of types. This category only includes appraisals in which at least two different types were combined, for example some health states were informed by a literature source and others by statistical mapping (for example HST21). If two different literature sources were used, this was not considered in this category but rather grouped in the literature category. Further, a combination of types also included indications for which different types of utilities were proposed by the manufacturer and the EAG, and the committee accepted utilities in-between both approaches [HST11, TA748] or from both approaches [TA343] (see S6 for further detail).

### Carer HRQoL

**Fig. 2:**
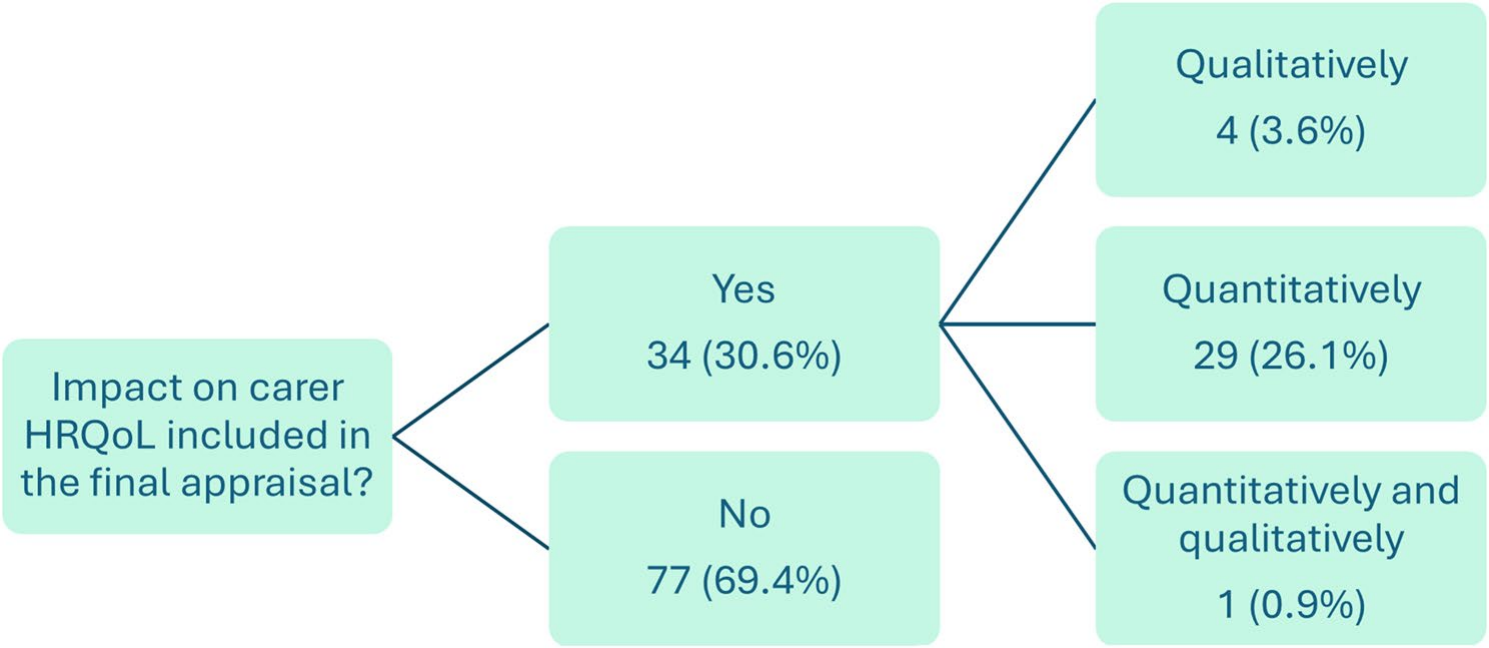
Overview of approaches for the consideration of carer HRQoL in the final appraisal (*n* = 111) HRQoL = health-related quality of life

The impact of carer HRQoL was included in the final appraisal in 34 (30.6%) indications (Fig. 2). Of these, carer HRQoL was included quantitatively in 26.1% of indications. In a further 3.6% of indications, the committee considered the health effects on carers qualitatively as it did not accept the initially proposed quantitative modelling approach. In one appraisal (burosumab for X-linked hypophosphatemia [HST8]), the manufacturer used a carer disutility value from a study of individuals with limited mobility, but the committee questioned whether the chosen value was appropriate for carers for people with X-linked hypophosphatemia, eventually considering the health effect on carers both quantitatively and qualitatively [45].

### Quantitative consideration of carer HRQoL

The majority of appraisals that accounted for the health effects of carers quantitatively used a carer disutility approach. This approach assigns disutility values for carers to each patient health state. This approach has been criticised because it typically assumes that carers have the same HRQoL as the general population after the patient’s death [46]. However, this issue can partly be addressed by adding a bereavement impact for carers, usually in form of an additional disutility [46]. In general, excluding QALY gains accrued by carers after the patient’s death means valuing the HRQoL of carers of surviving patients, but not that of bereaved carers [47]. Thus, inclusion or exclusion of carer’s HRQoL after the patient’s death requires a normative judgement.

In this dataset, bereavement disutility appears to have been accepted in 3 indications: in the appraisal of risdiplam for SMA type 1 [TA755 [1]], a bereavement disutility of 0.04 from the point of mean overall survival was included [48, 49]; likewise, in the appraisal of eladocagene exuparvovec for aromatic L‑amino acid decarboxylase deficiency [HST26] a similar disutility of 0.037 from the same source was applied [40]; and in the appraisal of velmanase alfa for alpha-mannosidosis [HST29] it was assumed that patients in the short-term end-stage health state die within four weeks and a disutility was applied for one year to account for carer bereavement [50]. In seven other indications, bereavement effects were either included in a scenario analyses or in the company base case but not in the final economic model [HST7 [51], HST22 [52, 53], HST23 (1) [54, 55], HST23 (2) [56], TA588 (1) and TA588 (2) [57, 58], TA755 (2) [47].

The number of carers also varied across indications (Table 2). In some indications, the number of carers was health state dependent, typically with carer burden increasing in more severe health states. For example, in the appraisal of atidarsagene autotemcel for metachromatic leukodystrophy (MLD) [HST18] it was assumed that an increasing number of carers were needed from health state 1 (0.5 carers) to health state 6 (2 carers) [59]. Other indications assumed an average of carers across all health states. For example, appraisals for cannabidiol assumed 1.8 carers were needed [TA614 [60], TA615 [61], TA873 [62]]. We classified two appraisals as ‘other’ because the mean number of carers was redacted [HST25] [63] or because the number of carers was dependent on patient age, with two carers assumed for children and one carer for adults [TA804] [64].

**Table 2:** Number of carers included in economic models (*n* = 29)

Number of carers	Number of indications (%)
1 carer	8 (27.6)
More than 1 carer	10 (34.4)
Health state dependent	9 (31.0)
Other	2 (6.9)

### Types of carer utility values

**Fig. 3:**
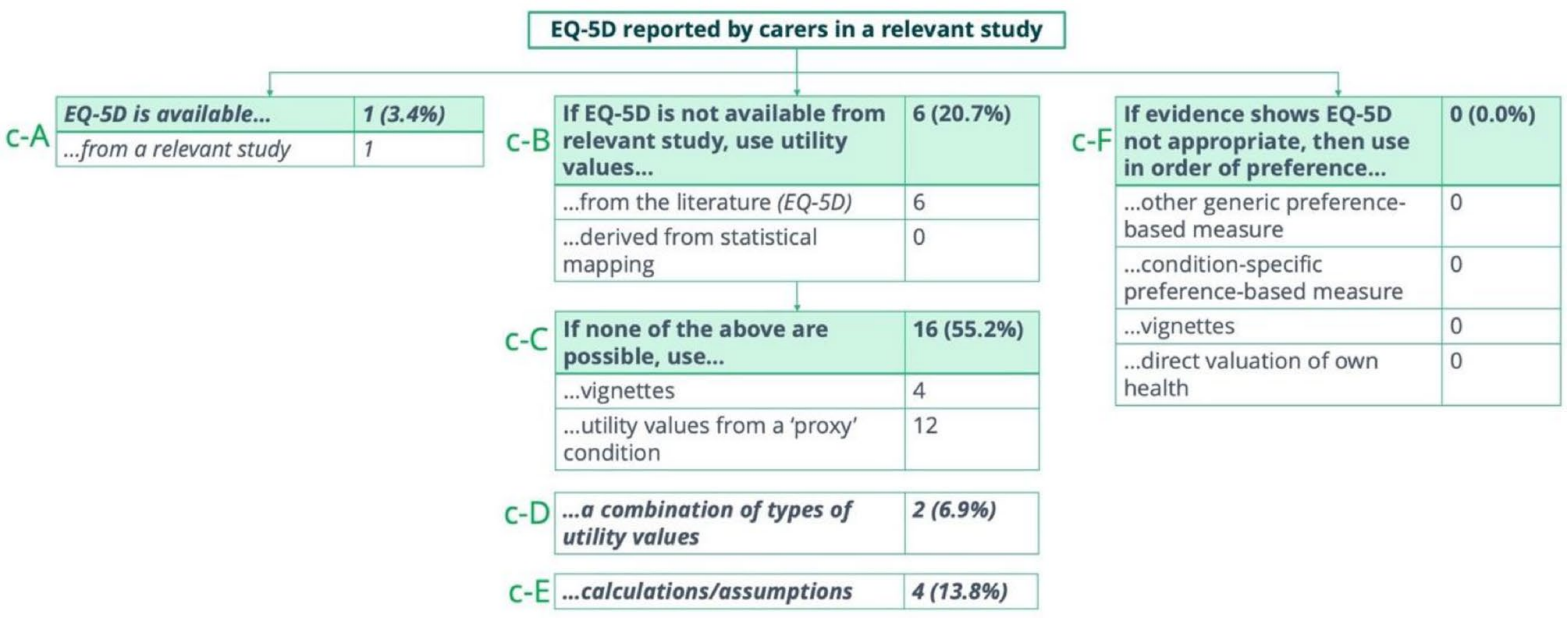
Types of carer utilities quantitatively included in the final appraisal (*n* = 29) notes: categories are labelled c-A to c-F, where “c” denotes carer utilities and letters (A–F) are used for distinction. Categories shown in italics were added by the authors. Categories c-D and c-E fall outside the NICE hierarchy

A range of approaches were used to model carer HRQoL. Overall, for most indications in which carer HRQoL was included quantitatively in the final appraisal, EQ-5D data was not available from a relevant study (categories c-B to c-E). Only in one indication (3.4%), was EQ-5D available and used (category c-A). There was no indication for which evidence showed that EQ-5D reported by carers in the relevant study was not appropriate (category c-F) (Fig. 3). Where EQ-5D was not available from a relevant study, manufacturers derived carer HRQoL from EQ-5D reported in the literature (20.7%), vignette studies or proxy conditions (55.2%), a combination of types of utility values (6.9%), or calculations/assumptions (13.8%) (Fig. 3). Categories c-D and c-E fall outside the NICE hierarchy.

### Where EQ-5D is available from a relevant study

Carer HRQoL data was obtained from a relevant study only in the appraisal of fenfluramine for seizures associated with Dravet syndrome [TA808]. In this appraisal, EQ-5D-5 L data were collected in two randomised controlled trials (study 1 and study 1504) and mapped onto EQ-5D-3 L using van Hout et al. [65, 66].

### Where EQ-5D is not available from a relevant study

Where EQ-5D was not available from a relevant study, carer HRQoL data was derived mostly from the published literature, vignettes or proxy conditions. Regarding the use of vignettes, we found that, similar to the studies designed to derive patient utilities, the vignettes used to evaluate carer HRQoL were not valued via the EQ-5D. This was not in line with NICE’s hierarchy of preferred HRQoL methods. For example, in the appraisal of birch bark extract for treating epidermolysis bullosa [HST28], the manufacturer commissioned an online cross-sectional study to elicit carer HRQoL but did not believe the results to be robust due to its small sample size [67]. Eventually the manufacturer derived carer HRQoL from vignettes evaluated by members of the general public using the Time Trade-Off method [68].

Importantly, we found that utility values from proxy conditions were used for many indications. Proxy conditions included multiple sclerosis [HST9, HST16, HST19, HST26, HST29], Alzheimer’s disease [HST10], meningitis [HST11], lipodystrophy and new musculoskeletal conditions [HST13], spinal muscular atrophy and moderate to severe atopic dermatitis [HST17], activity limitations [HST20], Duchenne muscular dystrophy [HST23 (2)], and stroke [TA667] (see S7, Table 8 for further detail).

Moreover, the impact on carer HRQoL was sometimes derived from a combination of approaches. For example, in the appraisal of cerliponase alfa for acquired thrombotic thrombocytopenic purpura [HST12], the manufacturer derived the disutility value for one health state from the literature, relied on expert input to inform the values for additional two health states, and derived the values for remaining health states based on a range of assumptions (see S7, Table 8 for further detail).

Finally, carer disutility values used in the appraisal of health technologies indicated for spinal muscular atrophy (nusinersen and risdiplam) [TA588 (1), TA588 (2)], TA755 (1), TA755 (2)] were calculated as a range based on different sources and assumptions. Here, the manufacturers estimated a carer disutility based on a range defined by the average utility from Spanish carers in López-Bastida et al. [69] and the general population EQ-5D score.

### Qualitative consideration of carer HRQoL

In those indications in which the committee considered the health effects on carers qualitatively, the initial modelling approach for carer HRQoL impacts by the manufacturer was not accepted. For example, in the appraisal of asfotase alfa for hypophosphatasia with perinatal or infantile onset [HST23 (1)] the manufacturer used data from a study evaluating the HRQoL impact for carers for people with Duchenne muscular dystrophy [56]. This approach was not accepted by the committee because it produced counter-intuitive outcomes, whereby increased patient survival resulted in a lower QALY gain as carers experienced disutility for a longer period [55]. However, the committee accepted the use of a carer disutility derived from the appraisal of ataluren for treating Duchenne muscular dystrophy (HST3) for patients with juvenile-onset hypophosphatasia, as this issue of counter-intuitive outcomes did not apply in that context [55].

In the reappraisal of ataluren for Duchenne muscular dystrophy [HST22] health gains by carers were modelled using an ‘absolute’ approach to measure carer QALYs in the company base case [46]. This approach links QALYs of carers to patient survival because the probability of patients being in specific health states is multiplied by a carer utility [46]. The committee did not accept this approach due to the problematic assumption that after death of the patient carers had zero QALYs (or they were not valued by society) [53]. Given that the disutility approach suggested by the EAG resulted in a higher incremental cost-effectiveness ratio because of the negative carer QALY gain, the committee eventually considered the impact on carer HRQoL qualitatively [53].

Lastly, in the appraisal of mogamulizumab for Sézary syndrome and mycosis fungoides [TA754] the manufacturer estimated carer utility values based on the results of its vignette study and including several assumptions [70]. The committee did not accept this approach because the estimated QALY gain of carers was unrealistically large, the vignette study used to estimate utilities did not correspond to NICE’s reference case of measuring HRQoL using the EQ-5D, and the unvalidated assumption that the difference between carer utilities for specific health states was equal to the difference for patients in the trial [71]. Eventually, the committee considered the health effect on carers qualitatively [71].

## Discussion

### Patient utilities

Our study found that, in over half (59.5%) of the RDT indications analysed, EQ-5D data was not used to inform patient utilities in in the final economic model. Therefore, in the majority of appraisals for RDTs, utilities in economic models were not based on NICE’s preferred method of using EQ-5D data from a relevant study. This finding suggests that, given the NICE hierarchy of preferred HRQoL methods, patient and carer HRQoL was not captured very well in RDT appraisals.

There is also heterogeneity in the approaches used to generate patient utilities. This is similar to findings from previous studies [23, 24]. A combination of approaches was used to inform patient utilities in the final appraisal in approximately one-third (29.7%) of indications. This highlights that the NICE hierarchy viewed as a taxonomy of possible approaches does not accommodate some of the approaches used in RDT appraisals very well.

The frequent absence of EQ-5D data derived from a relevant study, several indications where EQ-5D was considered inappropriate, and the combination of different sources of patient utilities within an appraisal, likely reflect the poor evidence base for many RDTs. It could also be seen as a violation of NICE’s preferred methods. However, the low proportion of negative recommendations for RDTs (8.1%) suggests that NICE adopts a more flexible approach in accepting available evidence for RDTs. This flexibility is further supported by the fact that 76.6% (85/111) of the final appraisal or evaluation documents for the indications in our data set discussed approaches or uncertainties around patient utilities, suggesting that these issues were of some influence on NICE’s final recommendation. Most RDTs are evaluated under the TA process, which is described as adaptable to a wide range of technologies and conditions, including rare diseases [72]. Although different criteria apply to a subset of RDTs assessed under the HST guidance process [73], the large number of positive recommendations alongside the use of different types of patient utilities suggests that NICE has a flexible approach to utilities for RDTs.

### Carer utilities

Regarding carer HRQoL, we found that the use of proxy conditions to derive carer disutility values is predominant in appraisals in which EQ-5D was not available from a relevant study, confirming findings from Looby et al. [23], and likely reflecting the lack of data on carer burden for rare disease patients [74]. In addition, some appraisals used calculations and assumptions or a combination of types to inform the impact of carer HRQoL which is not aligned with NICE’s preferred methods. Furthermore, the methodological challenges involved in modelling the impact on carers’ HRQoL, and the resulting uncertainty in some appraisals, led the committee to consider the HRQoL of carers qualitatively in the final appraisal, rather than quantifying it in the economic model. This also suggests that NICE uses different ways to handle uncertainty in relation to carer HRQoL across appraisals.

In terms of modelling considerations, we found that a small number of indications included a disutility to reflect the impact of bereavement on carers in the final appraisal. NICE’s methods and process manual does not include guidance with regards to the inclusion of bereavement effects, and a report of a NICE task and finish group stated that methods for quantifying the effect of bereavement were not well developed [75]. Further, there seems no dominant approach taken regarding the number of carers modelled, with the number of carers depending on the severity of the patient health state, the assumption of one carer across all health states, or the assumption of more than one carer, often calculated as an average number, across health states. This differs from the findings of the earlier review of Pennington [21], in which the number of carers was assumed to be 1 in most of the appraisals analysed. This may be due to different criteria for selecting appraisals and the time period considered (we considered RDT appraisals between 2011 and 2023 whereas Pennington [21] analysed all NICE appraisals between 2000 and 2019). Both issues, the number of carers modelled and the consideration of bereavement, have also been identified as challenges when modelling carer HRQoL by Pennington and Al-Janabi [76]. Overall, our findings highlight the need for more guidance on incorporating carer HRQoL into economic models, and for more evidence on the impact of caring for rare disease patients on carer HRQoL.

### Implications for NICE’s HRQoL hierarchy

Overall, NICE’s hierarchy of preferred HRQoL methods seems a useful guide for manufacturers for making submissions, however by itself it is not ensuring that appraisals are done on a consistent basis using the preferred methods. NICE’s hierarchy offers some flexibility for manufacturers, and this analysis has shown that in practice this flexibility extends to situations which are not explicitly covered by the hierarchy, particularly when different types of utility values are combined. This may be appropriate in the context of RDTs where the available evidence is often limited. However, adopting a more flexible approach to the acceptance of different types of utility values can allow submissions to rely on evidence that is less robust than the methods typically preferred by NICE. While this flexibility can help to expand the evidence base, it also reduces the consistency and comparability of HRQoL evidence across appraisals. Stricter methodological requirements may support more consistent decision making, but risk excluding potentially relevant evidence, whereas greater flexibility can increase the amount of available evidence at the expense of comparability between appraisals.

### Limitations

This study has several limitations. First, the analysis focused on approaches taken and assumptions made in the final economic model that was used by the committee to inform its reimbursement decision. As such, this analysis did not necessarily capture all assumptions made in the manufacturer’s baseline model, throughout the appraisal, or in sensitivity analyses. Second, one difficulty was to determine cases in which patient utilities were informed by proxy conditions. This was largely due to the challenge of determining what counts as a ‘proxy’. In the absence of utilities specific to the population under review, some appraisals used utilities derived from people with different, though potentially related, conditions without explicitly referring to them as proxy conditions. For example, in the appraisal of cabozantinib for medullary thyroid cancer, utilities from a study with people with differentiated thyroid cancer were used because values specific to people with medullary thyroid cancer were not available ([TA516] [77]), although this was not explicitly referred to as a proxy condition in the committee papers. Ultimately, we categorised one appraisal (HST27) as consistent with the NICE hierarchy’s use of proxy patient utilities, as utilities derived from a condition explicitly described as a proxy were applied to all health states. In the supplementary material, we highlight appraisals that used proxy values for some health states, but this selection did not include indications in which utilities were derived from people with different, though potentially related, conditions without explicitly referring to them as proxy conditions. While this reflects a partly subjective analytical choice, it also highlights the difficulty of defining a ‘proxy’ condition or utility and the absence of a definition by NICE. Third, another difficulty was to determine with certainty which appraisals would pertain to categories p-G to p-I and c-F (‘evidence shows that EQ-5D was not appropriate’) because references to the inappropriateness of the EQ-5D or evidence to demonstrate this were not consistently reported across appraisals. We chose to categorise indications into this group when the manufacturer stated that EQ-5D data collected in the relevant study was not appropriate, and only if this was not dismissed by the committee, which may be subjective but which we considered to be a pragmatic analytical choice. Fourth, determining in how many appraisals the QALY gain was due to improvement in HRQoL as opposed to other factors and thus the relative importance of assumptions around patient utilities for the NICE recommendation was not possible in this study and remains an area for further research. Finally, we reviewed and described approaches used to incorporate carer HRQoL in economic models used for RDTs appraised by NICE. While we refer to some methodological challenges, a more detailed review of methodological challenges and potential alternatives to model carer HRQoL is provided elsewhere [76], including a research agenda to improve understanding of carer HRQoL in rare diseases [74].

## Conclusion

This study systematically analysed the approaches used to model patient and carer utilities in NICE appraisals of RDTs. It found heterogeneity in the approaches used to model patient and carer HRQoL. Our findings suggest that, given the NICE hierarchy of preferred HRQoL methods, patient and carer HRQoL was not very well captured in RDT appraisals. If the NICE hierarchy is viewed as a taxonomy of possible approaches, our findings also suggest that it does not accommodate some approaches used to estimate HRQoL in RDT appraisals. These issues raise questions about the consistency with which HRQoL is modelled in appraisals and reflect the challenges of generating robust HRQoL evidence for RDTs. The findings also highlight the need for more guidance on incorporating carer HRQoL into economic models, and for more evidence on how caring for rare disease patients affects carer HRQoL.

## Electronic supplementary material

Below is the link to the electronic supplementary material.

Supplementary material 1

## Data Availability

Appraisal documents issued by the National Institute for Health and Care Excellence are publicly available on their website: [https://www.nice.org.uk/guidance/published?ngt=Highly+specialised+technologies+guidance&ngt=Technology+appraisal+guidance].
