# Supplementary material for: Approaches used to model patient and carer health-related quality of life in economic models of rare disease treatments in NICE appraisals
Source: Health Qual Life Outcomes. 2026 Apr 13;24:71. doi: 10.1186/s12955-026-02532-w (PMC13185199; doi:10.1186/s12955-026-02532-w)
Supplement: Supplementary file 1 — Supplementary material 1 [file 12955_2026_2532_MOESM1_ESM.pdf]

Approaches to model patient and carer health-related quality of life in economic models of rare disease treatments in NICE appraisals

Supplementary material

S1 NICE's hierarchy of preferred HRQoL methods 2

S2 Appraisal selection 4

S3 List of analysed appraisals 6

S4 Therapeutic area 17

S5 Patient health state utility values 18

S6 Carer health-related quality of life 26

S1 List of abbreviations

Table 1: Abbreviations

EAG	Evidence Assessment Group
EQ-5D	EuroQoL-5 Dimension
HST	Highly Specialised Technology appraisal guidance
HSUV	Health state utility value
HTA	Health Technology Assessment
HRQoL	Health-related quality of life
MHRA	Medicines & Healthcare products Regulatory Agency
NICE	National Institute for Health and Care Excellence
NHS	National Health Service
QALY	Quality-adjusted life year
RDT	Rare disease treatment
TA	Technology Appraisal guidance
UK	United Kingdom

S2 NICE’s hierarchy of preferred HRQoL methods

Figure 1: Reproduction of NICE's hierarchy of preferred health-related quality of life methods according to the NICE manual

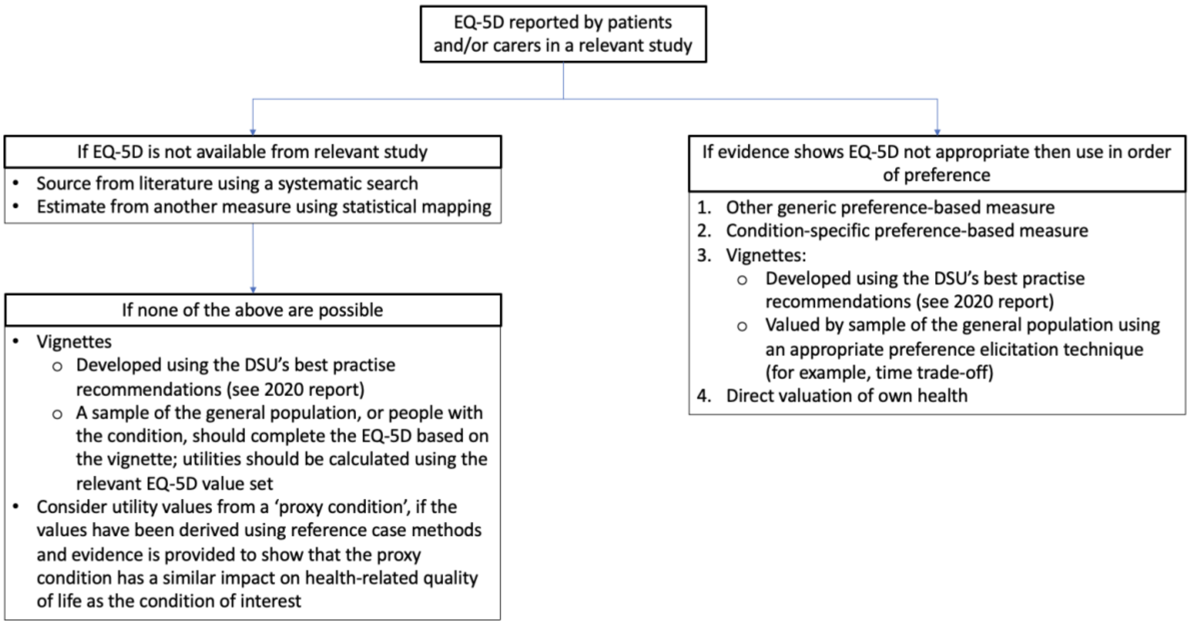

Source: Reproduced from NICE (1).

© NICE [2025]. NICE health technology evaluations: the manual [PMG36], p.200. Available from https://www.nice.org.uk/process/pmg36. All rights reserved. Subject to notice of rights.

S3 Appraisal selection

The guidance database of the National Institute for Health and Care Excellence (NICE) in England does not allow to filter for rare disease treatments (RDTs) but provides a filter to select appraisals conducted under the Technology Appraisal (TA) and the Highly Specialised Technology (HST) appraisal guidance processes. All completed appraisals published under the TA guidance up to the end of December 2023 were recorded, returning 943 appraisals. From these, 836 appraisals were excluded because they were published before the year 2011 (n=212) or because they were non-RDT appraisals (n=624). The Orphan Register published by the Medicines & Healthcare products Regulatory Agency was used to determine whether a health technology was an RDT. If an appraisal was available for a health technology listed in the Orphan Register, it was considered an RDT appraisal. From the identified 107 RDT appraisals, 32 appraisals were excluded because they have been terminated, withdrawn, updated and replaced, appraised multiple technology appraisals, or were cost comparisons, resulting in 75 appraisals from the TA guidance process. As this paper focused on analysing features of economic models, this study also excluded indications for which no economic model was submitted (n=2). This resulted in 75 RDT appraisals, including 55 active substances and 82 indications.

With regard to the HST guidance, 29 appraisals were completed and published within the selected timeframe. After excluding three appraisals because they have been updated and replaced, 26 appraisals were included from the HST guidance process.

In total, 101 appraisals were included in the dataset. These corresponded to 80 active substances and 111 indications. Figure 2 shows a flowchart describing the selection process for the appraisals from the NICE guidance database.

Figure 2: Appraisal selection – screening of NICE guidance database

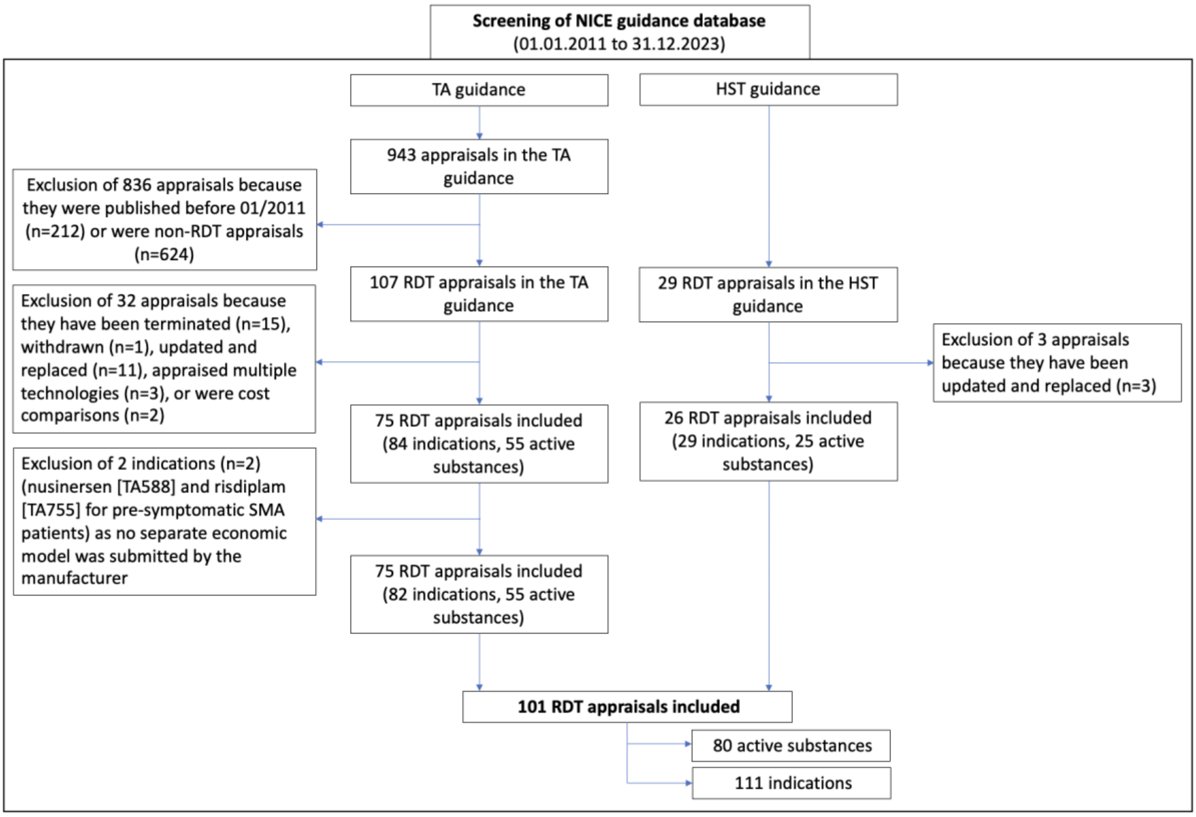

S4 List of analysed appraisals

Table 2: Overview of analysed appraisals (n=101) and indications (n=111)

ID	Active substance	Brand name	Indication	Recommendation (NICE)	Link to NICE website
HST27	afamelanotide	scenesse	erythropoietic protoporphyria	not recommended	https://www.nice.org.uk/guidance/hst27
TA813	asciminib	scemblix	chronic myeloid leukaemia, Ph+, after ≥ 2 tyrosine kinase inhibitors	recommended	https://www.nice.org.uk/guidance/ta813
HST23 (1)	asfotase alfa (1)	strensiq	perinatal onset (symptoms started before or at birth) or infantile onset (symptoms started between ages of 0-6 months)	optimised	https://www.nice.org.uk/guidance/hst23
HST23 (2)	asfotase alfa (2)	strensiq	juvenile onset (symptoms started between 6 months - 17 years)	optimised	
HST22	ataluren	translarna	Duchenne muscular dystrophy (≥ 2 y/o)	recommended	https://www.nice.org.uk/guidance/hst22
HST18 (1)	atidarsagene autotemcel (1)	libmeldy	children with late infantile (LI) or early juvenile (EJ) forms of metachromatic leukodystrophy (MLD) without clinical manifestation of the disease	recommended	https://www.nice.org.uk/guidance/hst18
HST18 (2)	atidarsagene autotemcel (2)	libmeldy	children with the early juvenile (EJ) form of metachromatic leukodystrophy (MLD) with early clinical manifestation of the disease, but who are still able to walk independently, before the onset of cognitive deterioration	recommended	
TA825	avacopan	tavneos	with a cyclophosphamide or rituximab regimen; severe active granulomatosis with polyangiitis or microscopic polyangiitis	recommended	https://www.nice.org.uk/guidance/ta825
TA872	axicabtagene ciloleucel (1)	yescarta	diffuse or primary mediastinal large B-cell lymphoma after at least 2 systemic therapies	recommended	https://www.nice.org.uk/guidance/ta872
TA894	axicabtagene ciloleucel (2)	yescarta	relapsed or refractory follicular lymphoma after 3 or more systemic treatments in adults	not recommended	https://www.nice.org.uk/guidance/ta894
TA895	axicabtagene ciloleucel (3)	yescarta	relapsed or refractory diffuse large B-cell lymphoma after first-line chemoimmunotherapy	recommended (CDF)	https://www.nice.org.uk/guidance/ta895

Table 2: Overview of analysed appraisals (n=101) and indications (n=111)

ID	Active substance	Brand name	Indication	Recommendation (NICE)	Link to NICE website
HST28	birk barch extract	filsuvez	partial thickness wounds associated with dystrophic and junctional epidermolysis bullosa in people aged 6 months and over	recommended	https://www.nice.org.uk/guidance/hst28
TA450	blinatumomab (1)	blincyto	Philadelphia-chromosome-negative acute lymphoblastic leukaemia	recommended	https://www.nice.org.uk/guidance/ta450
TA589	blinatumomab (2)	blincyto	acute lymphoblastic leukaemia in remission with minimal residual (MRD) disease activity	optimised	https://www.nice.org.uk/guidance/ta589
TA524 (1)	brentuximab vedotin (1)	adcetris	CD30-positive Hodgkin lymphoma after an autologous stem cell transplant	recommended	https://www.nice.org.uk/guidance/ta524
TA524 (2)	brentuximab vedotin (2)	adcetris	CD30-positive Hodgkin lymphoma in adults with increased risk of disease relapse or progression after autologous stem cell transplant	not recommended	
TA524 (3)	brentuximab vedotin (3)	adcetris	CD30-positive Hodgkin lymphoma after at least 2 previous therapies when autologous stem cell transplant or multi-agent chemotherapy are not suitable	recommended	
TA478	brentuximab vedotin (4)	adcetris	relapsed or refractory systemic anaplastic large cell lymphoma, only if patients have an Eastern Cooperative Oncology Group (ECOG) performance status of 0 or 1	optimised	https://www.nice.org.uk/guidance/ta478
TA577	brentuximab vedotin (5)	adcetris	CD30-positive cutaneous T-cell lymphoma, and only if patients have mycosis fungoides stage IIB or over, primary cutaneous anaplastic large cell lymphoma or Sezary syndrome	optimised	https://www.nice.org.uk/guidance/ta577
TA641	brentuximab vedotin (6)	adcetris	with cyclophosphamide, doxorubicin and prednisone (CHP); untreated systemic anaplastic large cell lymphoma	recommended	https://www.nice.org.uk/guidance/ta641
TA677	brexucabtagene autoleucel (1)	tecartus	relapsed or refractory mantle cell lymphoma after 2 or more lines of systemic therapy including a Bruton's tyrosine kinase (BTK) inhibitor	recommended (CDF)	https://www.nice.org.uk/guidance/ta677

Table 2: Overview of analysed appraisals (n=101) and indications (n=111)

ID	Active substance	Brand name	Indication	Recommendation (NICE)	Link to NICE website
TA893	brexucabtagene autoleucel (2)	tecartus	relapsed or refractory B-cell acute lymphoblastic leukaemia in people 26 years and over	recommended (CDF)	https://www.nice.org.uk/guidance/ta893
TA708	budesonide (1)	jorveza	Inducing remission of eosinophilic oesophagitis	recommended	https://www.nice.org.uk/guidance/ta708
TA937	budesonide (2)	Kinpeygo	primary IgA nephropathy	optimised	https://www.nice.org.uk/guidance/ta937
TA896	bulevirtide	hepcludex	chronic hepatitis D in adults, only if there is evidence of significant fibrosis and their hepatitis has not responded to peginterferon alfa-2a or they cannot have interferon-based therapy	optimised	https://www.nice.org.uk/guidance/ta896
HST8	burosumab	crysvita	X-linked hypophosphatemia in children (>1 y/o) and young people	recommended	https://www.nice.org.uk/guidance/hst8
TA516	cabozantinib	cometriq	medullary thyroid cancer in adults with unresectable, locally advanced or metastatic disease	recommended	https://www.nice.org.uk/guidance/ta516
TA614	cannabidiol (1)	epidyolex	with clobazam; seizures associated with Dravet syndrome (≥ 2 y/o)	optimised	https://www.nice.org.uk/guidance/ta614
TA615	cannabidiol (2)	epidyolex	with clobazam; seizures associated with Lennox-Gastaut syndrome (≥ 2 y/o)	optimised	https://www.nice.org.uk/guidance/ta615
TA873	cannabidiol (3)	epidyolex	seizures caused by tuberous sclerosis complex (≥ 2 y/o), only if their seizures are not controlled well enough by 2 or more antiseizure medications (either used alone or in combination) or these treatments were not tolerated, and seizure frequency is checked every 6 months, and cannabidiol is stopped if the frequency has not fallen by at least 30% compared with the 6 months before starting treatment	optimised	https://www.nice.org.uk/guidance/ta873
TA667	caplacizumab	cablivi	acquired thrombotic thrombocytopenic purpura (aTTP) (≥ 12 y/o) who weigh at least 40kg	recommended	https://www.nice.org.uk/guidance/ta667

Table 2: Overview of analysed appraisals (n=101) and indications (n=111)

ID	Active substance	Brand name	Indication	Recommendation (NICE)	Link to NICE website
TA695	carfilzomib (1)	kyprolis	with dexamethasone and lenalidomide; multiple myeloma only if patients have received at least one prior therapy, which included bortezomib	optimised	https://www.nice.org.uk/guidance/ta695
TA657	carfilzomib (2)	kyprolis	with dexamethasone; multiple myeloma only if patients have received at least one prior therapy	optimised	https://www.nice.org.uk/guidance/ta657
HST12	cerliponase alfa	brineura	neuronal ceroid lipofuscinosis type 2 (CLN2)	recommended (MAA)	https://www.nice.org.uk/guidance/hst12
TA720	chlormethine	ledaga	mycosis fungoides-type cutaneous T-cell lymphoma	optimised	https://www.nice.org.uk/guidance/ta720
TA912	cipaglucosidase alfa	pombiliti	with miglustat; late-onset Pompe disease	recommended	https://www.nice.org.uk/guidance/ta912
TA743	crizanlizumab	adakveo	Preventing recurrent sickle cell crises (vaso-occlusive crises) in sickle cell disease (≥16 y/o)	optimised (MAA)	https://www.nice.org.uk/guidance/ta743
TA763	daratumumab (1)	darzalex	in combination with bortezomib, thalidomide and dexamethasone; untreated multiple myeloma when a stem cell transplant is suitable	recommended	https://www.nice.org.uk/guidance/ta763
TA783	daratumumab (2)	darzalex	monotherapy; relapsed and refractory multiple myeloma, only if patients have daratumumab after 3 treatments	optimised	https://www.nice.org.uk/guidance/ta783
TA897	daratumumab (3)	darzalex	with bortezomib and dexamethasone; previously treated multiple myeloma, only if they had only 1 previous treatment and it included lenalidomide or lenalidomide is unsuitable as a second-line treatment	optimised	https://www.nice.org.uk/guidance/ta897
TA917	daratumumab (4)	darzalex	multiple myeloma (newly diagnosed), patients unsuitable for autologous stem cell transplant, combination with lenalidomide and dexamethasone	recommended	https://www.nice.org.uk/guidance/ta917
TA556	darvadstrocel	alofisel	previously treated complex perianal fistulas in adults with non-active or mildly active luminal Crohn's disease	not recommended	https://www.nice.org.uk/guidance/ta556
TA552	daunorubicin/cytarabine	vyxeos	untreated acute myeloid leukaemia	recommended	https://www.nice.org.uk/guidance/ta552
TA538	dinutuxumab beta	qarziba	neuroblastoma	optimised	https://www.nice.org.uk/guidance/ta538
HST1	eculizumab	soliris	Atypical haemolytic uraemic syndrome (aHUS)	recommended	https://www.nice.org.uk/guidance/hst1

Table 2: Overview of analysed appraisals (n=101) and indications (n=111)

ID	Active substance	Brand name	Indication	Recommendation (NICE)	Link to NICE website
HST26	eladocagene exuparvovec	upstaza	aromatic L-amino acid decarboxylase (AADC) deficiency (≥ 18 months) with a clinical, molecular and genetically confirmed diagnosis of AADC deficiency with a severe phenotype	recommended	https://www.nice.org.uk/guidance/hst26
HST5	eliglustat	cerdelga	type 1 Gaucher disease	recommended	https://www.nice.org.uk/guidance/hst5
HST19	elosulfase alfa	vimizim	mucopolysaccharidosis type 4A	recommended	https://www.nice.org.uk/guidance/hst19
TA756	fedratinib	inrebic	disease-related splenomegaly or symptoms in myelofibrosis post-polycythaemia vera myelofibrosis or post-essential thrombocythemia myelofibrosis, only if patients previously had ruxolitinib	optimised (CDF)	https://www.nice.org.uk/guidance/ta756
TA808	fenfluramin	fintepla	seizures associated with Dravet syndrome (≥ 2 y/o), only if seizures have not been controlled after trying 2 or more antiseizure medicines, and the frequency of convulsive seizures is checked every 6 months, and fenfluramine is stopped if it has not fallen by at least 30% compared with the 6 months before starting treatment	optimised	https://www.nice.org.uk/guidance/ta808
TA545	gemtuzumab ozogamicin	mylotarg	with daunorubicin and cytarabine; Untreated de novo CD33-positive acute myeloid leukaemia except acute promyelocytic leukaemia for (≥ 15 y/o)	optimised	https://www.nice.org.uk/guidance/ta545
TA642	gilteritinib	xospata	relapsed or refractory FLT3-mutation-positive acute myeloid leukaemia (AML)	optimised	https://www.nice.org.uk/guidance/ta642
HST16	givosiran	givlaari	acute hepatic porphyria (≥ 12 y/o)	optimised	https://www.nice.org.uk/guidance/hst16
TA927	glofitamab		relapsed or refractory diffuse large B-cell lymphoma after 2 or more systemic treatments	recommended	https://www.nice.org.uk/guidance/ta927
TA467 (1)	Ex vivo expanded autologous human corneal epithelial cells containing stem cells	holoclar (1)	Limbal stem cell deficiency after eye burns for treating one eye	optimised	https://www.nice.org.uk/guidance/ta467

Table 2: Overview of analysed appraisals (n=101) and indications (n=111)

ID	Active substance	Brand name	Indication	Recommendation (NICE)	Link to NICE website
TA467 (2)	Ex vivo expanded autologous human corneal epithelial cells containing stem cells	holoclar (2)	Limbal stem cell deficiency after eye burns for treating both eyes	only in research	
TA809	imlifidase	idefirix	desensitisation treatment before kidney transplant for adults who are waiting for a kidney transplant from a deceased donor, are highly sensitised to human leukocyte antigens (HLA), have a positive crossmatch with the donor and are unlikely to have a transplant under the available kidney allocation system (including prioritisation programmes for highly sensitised people). It is only recommended if a maximum of 1 dose is given.	optimised	https://www.nice.org.uk/guidance/ta809
HST9	inotersen	tegsedi	stage 1 and stage 2 polyneuropathy in adults with hereditary transthyretin amyloidosis	recommended	https://www.nice.org.uk/guidance/hst9
TA541	inotuzumab ozogamicin	besponsa	relapsed or refractory CD22-positive B-cell precursor acute lymphoblastic leukaemia	recommended	https://www.nice.org.uk/guidance/ta541
TA440	irinotecan	onivyde pegylated liposomal	in combination with 5-fluorouracil and leucovorin; pancreatic cancer after gemcitabine	not recommended	https://www.nice.org.uk/guidance/ta440
TA870	ixazomib	ninlaro	with lenalidomide and dexamethasone; treating relapsed or refractory multiple myeloma, only if patients have had 2 or 3 lines of therapy	optimised	https://www.nice.org.uk/guidance/ta870
TA606	lanadelumab	takhzyro	preventing recurrent attacks of hereditary angioedema (≥ 12 y/o), only if patients are eligible for preventive C1-esterase inhibitor treatment, and the lowest dosing frequency is used in line with the summary of product characteristic	optimised	https://www.nice.org.uk/guidance/ta606

Table 2: Overview of analysed appraisals (n=101) and indications (n=111)

ID	Active substance	Brand name	Indication	Recommendation (NICE)	Link to NICE website
TA591	letermovir	prevymis	preventing cytomegalovirus (CMV) reactivation and disease after an allogeneic haematopoietic stem cell transplant (HSCT) in adults who are seropositive for CMV	recommended	https://www.nice.org.uk/guidance/ta591
HST25	lumasiran	oxlumo	primary hyperoxaluria type 1 (PH1)	recommended	https://www.nice.org.uk/guidance/hst25
TA266	mannitol	bronchitol	cystic fibrosis	optimised	https://www.nice.org.uk/guidance/ta266
TA860	maribavir	livtencity	refractory cytomegalovirus infection after a haematopoietic stem cell transplant or solid organ transplant	recommended	https://www.nice.org.uk/guidance/ta860
HST14 (1)	metreleptin (1)	myalepta	generalised lipodystrophy in adults and children (≥ 2 y/o)	recommended	https://www.nice.org.uk/guidance/hst14
HST14 (2)	metreleptin (2)	myalepta	partial lipodystrophy in adults and children (≥ 12 y/o), only if they have an HbA1c level above 58 mmol/mol (7.5%), or fasting triglycerides above 5.0 mmol/litre, or both	optimised	
TA748	mexiletine	namuscla	myotonia in non-dystrophic myotonic disorders	recommended	https://www.nice.org.uk/guidance/ta748
TA523	midostaurin (1)	rydapt	untreated acute myeloid leukaemia	recommended	https://www.nice.org.uk/guidance/ta523
TA728	midostaurin (2)	rydapt	advanced systemic mastocytosis	recommended	https://www.nice.org.uk/guidance/ta728
HST4	migalastat	galafold	fabry disease (≥ 16 y/o), only if enzyme replacement therapy (ERT) would otherwise be offered	optimised	https://www.nice.org.uk/guidance/hst4
TA754 (1)	mogamulizumab (1)	poteligeo	previously treated with at least 1 systemic treatment; Sezary syndrome	recommended	https://www.nice.org.uk/guidance/ta754
TA754 (2)	mogamulizumab (2)	poteligeo	mycosis fungoides, only if patients have stage 2B or above and they have had at least 2 systemic treatments	optimised	
TA892	mosunetuzumab	lunsumio	relapsed or refractory follicular lymphoma in adults who have had 2 or more systemic therapies	not recommended	https://www.nice.org.uk/guidance/ta892

Table 2: Overview of analysed appraisals (n=101) and indications (n=111)

ID	Active substance	Brand name	Indication	Recommendation (NICE)	Link to NICE website
TA673	niraparib (1)	zejula	maintenance treatment for advanced (FIGO stages 3 and 4) high-grade epithelial ovarian, fallopian tube or primary peritoneal cancer after response to first-line platinum-based chemotherapy	recommended (CDF)	https://www.nice.org.uk/guidance/ta673
TA784	niraparib (2)	zejula	maintenance treatment of relapsed, platinum-sensitive ovarian, fallopian tube and peritoneal cancer that has responded to the most recent course of platinum-based chemotherapy, only if patients have a BRCA mutation and have had 2 courses of platinum-based chemotherapy, or patients do not have a BRCA mutation and have had 2 or more courses of platinum-based chemotherapy	optimised	https://www.nice.org.uk/guidance/ta784
TA588 (1)	nusinersen (1)	spinraza	SMA Type 1	optimised (MAA)	https://www.nice.org.uk/guidance/ta588
TA588 (2)	nusinersen (2)	spinraza	SMA Type 2/3	optimised (MAA)	
TA443	cholic acid	ocaliva	primary biliary cholangitis	recommended	https://www.nice.org.uk/guidance/ta443
TA343	obinutuzumab (1)	gazyvaro	in combination with chlorambucil; untreated chronic lymphocytic leukaemia, only if bendamustine-based therapy is not suitable	optimised	https://www.nice.org.uk/guidance/ta343
TA513	obinutuzumab (2)	gazyvaro	untreated advanced follicular lymphoma, only if patients have a Follicular Lymphoma International Prognostic Index (FLIPI) score of 2 or more	optimised	https://www.nice.org.uk/guidance/ta513
TA629	obinutuzumab (3)	gazyvaro	with bendamustine; follicular lymphoma after rituximab	recommended	https://www.nice.org.uk/guidance/ta629
HST17	odevixibat	bylvay	progressive familial intrahepatic cholestasis (≥ 6 months)	recommended	https://www.nice.org.uk/guidance/hst17
HST15	onasemnogene abeparvovec (1)	zolgensma	type 1 SMA, only if patients are aged 6 months and younger they are aged 7 to 12 months, and their treatment is agreed by the national multidisciplinary team	optimised	https://www.nice.org.uk/guidance/hst15

Table 2: Overview of analysed appraisals (n=101) and indications (n=111)

ID	Active substance	Brand name	Indication	Recommendation (NICE)	Link to NICE website
HST24	onasemnogene abeparvovec (2)	zolgensma	pre-symptomatic SMA (≤ 12 months, up to 3 copies of the SMN2 gene)	optimised	https://www.nice.org.uk/guidance/hst24
TA380	panobinostat	farydak	in combination with bortezomib and dexamethasone; multiple myeloma after at least 2 previous treatments	recommended	https://www.nice.org.uk/guidance/ta380
HST10	patisiran	onpattro	hereditary transthyretin amyloidosis in adults with stage 1 and stage 2 polyneuropathy	recommended	https://www.nice.org.uk/guidance/hst10
TA778	pegcetacoplan	aspaveli	paroxysmal nocturnal haemoglobinuria	recommended	https://www.nice.org.uk/guidance/ta778
TA722	pemigatinib	pemazyre	relapsed or refractory advanced cholangiocarcinoma with FGFR2 fusion or rearrangement	recommended	https://www.nice.org.uk/guidance/ta722
TA504	pirfenidone	esbriet	idiopathic pulmonary fibrosis, only if the person has a forced vital capacity (FVC) between 50% and 80% predicted, and treatment is stopped if there is evidence of disease progression (an absolute decline of 10% or more in predicted FVC within any 12-month period).	optimised	https://www.nice.org.uk/guidance/ta504
TA649	polatuzumab vedotin (1)	polivy	with rituximab and bendamustine; Relapsed or refractory diffuse large B-cell lymphoma	recommended	https://www.nice.org.uk/guidance/ta649
TA874	polatuzumab vedotin (2)	polivy	with rituximab, cyclophosphamide, doxorubicin and prednisolone (R-CHP); untreated diffuse large B-cell lymphoma, only if patients have an International Prognostic Index (IPI) score of 2 to 5	optimised	https://www.nice.org.uk/guidance/ta874
TA427	pomalidomid	imnovid	in combination with low-dose dexamethasone; multiple myeloma, after 3 previous treatments including both lenalidomide and bortezomib	optimised	https://www.nice.org.uk/guidance/ta427

Table 2: Overview of analysed appraisals (n=101) and indications (n=111)

ID	Active substance	Brand name	Indication	Recommendation (NICE)	Link to NICE website
TA451 (1)	ponatinib (1)	iclusig	chronic myeloid leukaemia, only if the disease is resistant to dasatinib or nilotinib, or patients cannot tolerate dasatinib or nilotinib and for whom subsequent treatment with imatinib is not clinically appropriate or the T315I gene mutation is present	recommended	https://www.nice.org.uk/guidance/ta451
TA451 (2)	ponatinib (2)	iclusig	Philadelphia-chromosome-positive acute lymphoblastic leukaemia, only if the disease is resistant to dasatinib, patients cannot tolerate dasatinib and for whom subsequent treatment with imatinib is not clinically appropriate or the T315I gene mutation is present	recommended	
TA881	ripretinib	qinlock	gastrointestinal stromal tumour in adults after 3 or more treatments	not recommended	https://www.nice.org.uk/guidance/ta881
TA755 (1)	risdiplam (1)	evrysdi	Type 1 SMA	optimised (MAA)	https://www.nice.org.uk/guidance/ta755
TA755 (2)	risdiplam (2)	evrysdi	Type 2/3 SMA	optimised (MAA)	
HST20	selumetinib	koselugo	symptomatic and inoperable plexiform neurofibromas associated with type 1 neurofibromatosis (≥ 3 - 18 y/o)	recommended	https://www.nice.org.uk/guidance/hst20
HST21	setmelanotide	imcivree	obesity caused by LEPR or POMC deficiency (≥ 6 y/o)	recommended	https://www.nice.org.uk/guidance/hst21
HST7	autologous CD34+ enriched cell fraction that contains CD34+ cells transduced with retroviral vector that encodes for the human ADA cDNA sequence	strimvelis	adenosine deaminase deficiency–severe combined immunodeficiency	recommended	https://www.nice.org.uk/guidance/hst7
TA696	tafamidis	vyndaqel	hereditary transthyretin amyloidosis with cardiomyopathy (ATTR-CM)	not recommended	https://www.nice.org.uk/guidance/ta696
TA883	tafasitamab	minjuvi	with lenalidomide; relapsed or refractory diffuse large B-cell lymphoma in adults who cannot have an autologous stem cell transplant	not recommended	https://www.nice.org.uk/guidance/ta883

Table 2: Overview of analysed appraisals (n=101) and indications (n=111)

ID	Active substance	Brand name	Indication	Recommendation (NICE)	Link to NICE website
TA804	teduglutide	revestive	short bowel syndrome (≥ 1 y/o)	recommended	https://www.nice.org.uk/guidance/ta804
TA554	tisagenlecleucel (1)	kymriah	relapsed or refractory B-cell acute lymphoblastic leukaemia (≤ 25 y/o)	recommended (CDF)	https://www.nice.org.uk/guidance/ta554
TA567	tisagenlecleucel (2)	kymriah	relapsed or refractory diffuse large B-cell lymphoma after 2 or more systemic therapies	recommended (CDF)	https://www.nice.org.uk/guidance/ta567
TA640	treosulfan	trecondi	with fludarabine; malignant disease before allogeneic stem cell transplant	recommended	https://www.nice.org.uk/guidance/ta640
HST29	velmanase alfa	lamzede	alpha-mannosidosis in people under 18 years and in people who turn 18 while on treatment	optimised	https://www.nice.org.uk/guidance/hst29
HST13	volanesorsen	waylivra	familial chylomicronaemia syndrome	recommended	https://www.nice.org.uk/guidance/hst13
HST11	voretigen neparvovec	luxturna	inherited retinal dystrophies caused by RPE65 gene mutations	recommended	https://www.nice.org.uk/guidance/hst11

S5 Therapeutic area

Table 3: Therapeutic area of analysed RDT indications (n=111)

Therapeutic area	Number of indications (%)
Blood and immune system conditions	54 (48.6)
Cancer	8 (7.2)
Cardiovascular conditions	3 (2.7)
Cystic fibrosis	1 (0.9)
Diabetes and other endocrinal, nutritional and metabolic conditions	18 (16.2)
Digestive tract conditions	2 (1.8)
Eye conditions	3 (2.7)
Infections	3 (2.7)
Kidney conditions	3 (2.7)
Liver conditions	2 (1.8)
Musculoskeletal conditions	7 (6.3)
Neurological conditions	5 (4.5)
Respiratory conditions	1 (0.9)
Skin conditions	1 (0.9)

RDT = rare disease treatment

S6 Patient health state utility values

Table 4 gives an overview of appraisals in which EQ-5D data is available for patient health state utility values.

Table 4: Appraisals in which EQ-5D is available for patient health state utility values (n=38)

EQ-5D is available from…	**N (%)**	**Appraisals**
…a relevant study	*23 (20.7)*	**HST1** (EQ-5D from C08-002A/B and C08-003A/B)<br>**HST10** (EQ-5D (and COMPASS-31 and Norfolk QoL-DN) from APOLLO)<br>**HST19** (EQ-5D from managed access agreement; utility increment associated with an increase in 6MWT and in FVC outcomes for intervention arm based on Lampe et al. (2))<br>**HST25** (EQ-5D from ILLUMINATE-A; average from subgroup in ILLUMINATE-C for late CKD health states)<br>**HST28** (EQ-5D from EASE)<br>**TA427** (EQ-5D from MM-003; disutility for receiving intravenous or subcutaneous therapy based on previous NICE appraisals in small cell lung cancer (TA192, Erlotinib for NSCLC))<br>**TA591** (EQ-5D from PN001; disutility for long-term effects based on difference between mean utility of patients in PN001 at 48 weeks and the mean general population utilities; disutility due to GvHD from Pidala et al. (3))<br>**TA641** (EQ-5D from ECHELON-2)<br>**TA642** (EQ-5D from ADMIRAL; disutility for high-intensity chemotherapy from Wehler et al. (4))<br>**TA673** (EQ-5D from PRIMA)<br>**TA677** (EQ-5D from ZUMA-2)<br>**TA696** (EQ-5D from ATTRACT)<br>**TA783** (EQ-5D from MM-003)<br>**TA784** (EQ-5D from NOVA)<br>**TA860** (EQ-5D from SOLISTICE; decrements for graft failure sourced from manufacturer's vignette study)<br>**TA870** (EQ-5D from TMM1)<br>**TA872** (EQ-5D from ZUMA-1)<br>**TA881** (EQ-5D from INVICTUS)<br>**TA892** (EQ-5D from GO29781)<br>**TA893** (EQ-5D from ZUMA-3; utility multiplier to account for toxicity from previous therapies calculated from a ratio between the utility value reported in ZUMA-3 after infusion and before relapse and the general population)<br>**TA896** (EQ-5D from MYR301)<br>**TA897** (EQ-5D from ENDEAVOUR)<br>**TA917** (EQ-5D from MAIA)

Table 4: Appraisals in which EQ-5D is available for patient health state utility values (n=38)

EQ-5D is available from…	**N (%)**	**Appraisals**
…a relevant study for at least some of the health states (combination of types of utility values)	*15 (13.5)*	**HST29** (EQ-5D from rhLAMAN; UK MPS Society Survey)<br>**TA513** (EQ-5D from GALLIUM; values for early and late progressed state from Wild et al. (5) and 6))<br>**TA524 (3)** (EQ-5D from AETHERA for pre-relapse health states, HSUV for the PFS health state from Swinburn et al. (7), assumption that 5 years after starting treatment, HRQoL for people whose disease did not progress moves back to population norm, with a small utility decrement being applied)<br>**TA541** (EQ-5D from INO-VATE; value for progression sub-state from Aristides et al. (8); value for VOD assumed to be similar to that reported for acute liver failure prior to transplant; most appropriate post-transplant utility values between Kurosawa et al. (9) and general population post-transplant utility)<br>**TA554** (EQ-5D from ELIANA; survival utility post two years from Kelly et al. (10); disutility applied 3-12 months post stem cell transplant from Sung et al. (11) and Felder-Puig et al. (12); treatment disutility from Sung et al. (11); disutility due to Cytokine Release Syndrome based on ELIANA)<br>**TA577** (EQ-5D and Skindex-29 from ALCANZA; utilities for alloSCT health states from van Agthoven et al. (13) and end stage symptom care management from Swinburn et al. (7))<br>**TA589** (EQ-5D from BLAST; EORTC QLQ-C30 from TOWER mapped to EQ-5D using Longworth and Rowen (14); disutility due to HSCT from Kurosawa et al. (9); disutility to reflect long-term effects of exposure to radiotherapy, chemotherapy, and HSCT (based on half the difference between the average utility value for blinatumomab patients in the RFS state, off therapy, and with MRD response and the general population norm))<br>**TA629** (EQ-5D from GADOLIN (progression-free state); Wild et al. (5))<br>**TA754 (1) and TA754 (2)** (EQ-5D from MAVORIC; Swinburn et al. (7) (end of life) and van Agthoven et al. (13) (after stem cell transplant))<br>**TA763** (EQ-5D from CASSIOPEIA; TA311)<br>**TA813** (EQ-5D from ASCEMBL; TA451; Szabo et al. (15))<br>**TA825** (EQ-5D from ADVOCATE; TA623)<br>**TA895** (EQ-5D from ZUMA-7; pre-progression utilities from ZUMA-1 in TA559 representing a 3L+ population)<br>**TA912** (EQ-5D from PROPEL; vignettes)

Table 5 gives an overview of appraisals in which EQ-5D data was not available from a relevant study.

Table 5: Appraisals in which EQ-5D is not available from a relevant study (n=66)

If EQ-5D is not available from relevant study, use utility values…	N (%)	Appraisals
…from the literature (EQ-5D)	*12 (10.8)*	**HST4** (utility values from Rombach et al. (16); disutilities for complications based on proxy conditions; infusion disutilities from a discrete choice experiment from Lloyd et al. (17) (committee accepted the EAG's preferred analysis in which infusion disutilities were reduced by 50%))<br>**TA440** (TA476)<br>**TA443** (Younossi et al. (18) (HUI value for low and moderate risk states) and Wright et al. (19) (EQ-5D for all other states) used in TA330 on sofosbuvir for treating chronic hepatitis C (proxy condition))<br>**TA545** (TA399, Kurosawa et al. (9))<br>**TA606** (Nordenfelt et al. (20); utility increment for subcutaneous injections (vs IV administration) of the intervention from Jørgensen et al. (21))<br>**TA649** (TA559)<br>**TA708** (manufacturer proposes to use a disutility for gasto-oesophageal reflux disease (GORD) from a published literature source, while the EAG suggested alternative values from the literature for the condition in question (eosinophilic oesophagitis)) → the committee concluded that both approaches to utilities were suitable for decision making<br>**TA809** (Cooper et al. (22))<br>**TA874** (Sehn et al. (23))<br>**TA883** (TA559; disutility from subsequent CAR-T therapy for first two month of therapy from Lin et al. (24))<br>**TA894** (Wild et al. (5))<br>**TA937** (Cooper et al. (22))
…from the literature (non-EQ-5D)	*11 (9.9)*	**HST7** (vignettes evaluated by members of the public using the TTO method from Swinburn et al. (7); utility in first 6 months post procedure from Sung et al. (11))<br>**HST22** (HUI3 utility values obtained from a Delphi panel involving clinical experts taken from Landfeldt et al. (25))<br>**TA524 (1)** (vignettes evaluated by members of the public using the TTO method from Swinburn et al. (7))<br>**TA451 (1)** and **TA451 (2)** (vignettes evaluated by members of the public using the TTO method from Szabo et al. (15), the manufacturer assumed that the utilities reported by Szabo et al. (15) BP-CML were applicable for patients with Ph+ ALL)<br>**TA467 (1) and TA467 (2)** (value for visual acuity from Brown et al. (26); value for disfigurement from TA409 (using a utility value for cataracts as a proxy))<br>**TA478** (vignettes evaluated by members of the public using the TTO method from Swinburn et al. (7); utility decrement based on expert opinion to reflect that long term survivors may not regain full utility)<br>**TA516** (vignettes evaluated by members of the public using the TTO method from Fordham et al. (27))

Table 5: Appraisals in which EQ-5D is not available from a relevant study (n=66)

If EQ-5D is not available from relevant study, use utility values...	N (%)	Appraisals
		**TA524 (3)** (vignettes evaluated by members of the public using the TTO method from Swinburn et al. (7); utilities for stem cell transplant states from van Agthoven et al. (13))<br>**TA538** (reduction in general population utility estimate based on HUI3 utility values form Portwine et al. (28))
...derived from statistical mapping	11 (9.9)	**HST5** (SF-36 from GD-DS3 to EQ-5D using Brazier and Roberts (29); EAG assumption for increment for oral therapy over infusion therapy)<br>**TA450** (EORTC QLQ-C30 from TOWER to EQ-5D using Longworth et al. (30))<br>**TA504** (SGRQ from CAPACITY to EQ-5D using Freemantle et al. (31); disutilities for acute exacerbations based on TA397)<br>**TA567** (SF-36 from JULIET to EQ-5D using Rowen et al. (32); disutility for patients undergoing stem cell transplant from Guadagnolo et al. (33); disutility due to ICU stay based on JULIET)<br>**TA657** (EORTC QLQ-C30 from ENDEAVOUR and ASPIRE to EQ-5D using Proskorovsky et al. (34))<br>**TA695** (EORTC QLQ-C30 from ASPIRE to EQ-5D using Proskorovsky et al. (34))<br>**TA728** (SF-12 from D2201 to EQ-5D using Gray et al. (35); utility decrements to account for discomfort associated with subcutaneous or intravenous administration and the frequency of administration from Matza et al. (36) and Matza et al. (37))<br>**TA722** (EORTC QLQ-C30 from FIGHT 202 to EQ-5D using Longworth et al. (30); treatment administration disutility within the range of several studies (TA427, Jørgensen et al. (21), Paracha et al. (38))<br>**TA778** (EORTC QLQ-C30 from PEGASUS to EQ-5D using Longworth et al. (30); disutility to account for the effect of chelation therapy from Cherry et al. (39) and a frequent regular eculizumab infusion based on TA698)<br>**TA808** (PedsQL from Study 1 to EQ-5D using Khan et al. (40))<br>**TA927** (EORTC QLQ-C30 from NP30179 to EQ-5D using Proskorovsky et al. (34), assumption that people that do not progress for 3 years have a 10% utility decrement compared to the general population)
...vignettes	9 (8.1)	**HST8** (vignettes evaluated by clinical experts using EQ-5D)<br>**HST18 (1)** and **HST18 (2)** (manufacturer used vignettes evaluated by members of the public using the TTO method)<br>**HST20** (vignettes evaluated by members of the public using the TTO method)<br>**HST26** (manufacturer used vignettes evaluated by members of the public using the TTO method)<br>**TA556** (vignettes evaluated by members of the public using the TTO method)<br>**TA614** (vignettes evaluated by patients and/or caregivers using VAS)<br>**TA615** (vignettes evaluated by patients and/or caregivers using VAS)<br>**TA720** (vignettes evaluated by clinical experts using EQ-5D)
...utility values from a 'proxy' condition	1 (0.9)	**HST27** (utility values from confidential proxy condition)

Table 5: Appraisals in which EQ-5D is not available from a relevant study (n=66)

**If EQ-5D is not available from relevant study, use utility values…**	**N (%)**	**Appraisals**
…clinical expert input	3 (2.7)	**TA588 (1)** (López-Bastida et al. (41) (parent proxy) and Lloyd et al. (42) (vignettes valued by clinicians using EQ-5D) have limited face validity → manufacturer used estimates from clinical experts)<br>**TA588 (2)** (PedsQL from CHERISH to EQ-5D using Khan et al. (40) but preference-based utility estimates have limited face validity → manufacturer used estimates from clinical experts)<br>**TA755 (1)** (preference-based utility estimates have limited face validity → manufacturer used estimates from clinical experts in TA588; disutilities due to disease complications sourced from the SUNFISH trial and Lloyd et al. (42))
…generic measure	*1 (0.9)*	**TA743** (manufacturer mapped SF-36 data from SUSTAIN to EQ-5D UK utility values to calculate differential values for vaso-occlusive crises states, but resulting values were clinically implausible and the inappropriate assumption to account for complications and long-term organ damage through utility values of vaso-occlusive crises groups → committee accepts the Evidence Review Groups proposal of using a single utility value across vaso-occlusive crises health states based on weighted average from SF-36 data from SUSTAIN, with additional decrements applied for vaso-occlusive crises events and complications)
…a combination of sources	*18 (16.2)*	**HST9** (assumptions based on THAOS registry and Stewart et al. (43) reporting utilities according to Coutinho stages using a Brazilian value set) – *assumptions/calculations*<br>**HST11** (manufacturer used vignettes evaluated by clinical experts using EQ-5D, while the EAG proposed to use utility values from Rentz et al. (44) in which health states defined by 6 items of a disease specific HRQoL questionnaire (NEI VFQ-25) were evaluated by members of the public using the TTO method) → The committee concluded that the utility values fell between the ERG's preferred base case (Rentz et al. (44)) and the vignette-based EQ-5D values by the manufacturer) – *assumptions/calculations*<br>**HST14 (1) and HST14 (2)** (manufacturer considered EQ-5D domains not appropriate for the condition → value for pancreatitis taken from manufacturer's discrete choice experiment (DCE); additional utility differential based on the DCE to capture other symptoms not already accounted for; utility decrements for other organs taken from published sources and previous NICE appraisals of type 2 diabetes and fatty liver disease) – *DCE + literature*<br>**HST15** (estimates from clinical experts in TA588; Thompson et al. (45) reporting parent proxy EQ-5D values; additional utility gains based on Institute for Clinical and Economic Review (ICER) (46)) – *clinical experts + literature*<br>**HST17** (PedsQL from PEDFIC to EQ-5D using Khan et al. (40) (serum bile acid response and serum bile acid loss of response); parent-proxy PedsQL from Kamath et al. (47) to EQ-5D (response and loss of response to PEBD); disutility from Arseneau et al. (48) (effect of having a stoma bag); PedsQoL disutility for from Al-Uzri et al. (49) to EQ-5D (short stature); disutility from Kini et al. (50)

Table 5: Appraisals in which EQ-5D is not available from a relevant study (n=66)

If EQ-5D is not available from relevant study, use utility values…	N (%)	Appraisals
		(liver transplant); PedsQL disutility from Parmar et al. (51) to EQ-5D (post-liver transplant)) – *mapping + literature*<br>**HST21** (mapping for paediatric patients in BMI-Z health states: PedsQL from Riazi et al. (52) to EQ-5D using Khan et al. (40); published literature for paediatric patients in BMI health states; published literature for adult patients in BMI health states: Alsumali et al. (53) which collected data using the SF-12 and mapped values to EQ-5D; disutilities for comorbidities from Søltoft et al. (54) and Sullivan et al. (55)); utility modifier for hyperphagia based on manufacturer's vignette study was applied to HSUVs for BMI health states – *mapping + literature*<br>**HST24** (estimates from clinical experts in TA588; Thompson et al. (45) reporting parent proxy EQ-5D values) – *clinical experts + literature*<br>**TA266** (other generic preference-based measure (HUI2 values from DPM-CF-302); EQ-5D values for lung transplantation from Anyanwu et al. (56) and EQ-5D values for pulmonary exacerbations from 57)) – *other generic preference-based measure + literature*<br>**TA343** (vignettes evaluated by members of the public using the TTO method; utility value for PFS after first cycle of obinutuzumab based on assumption; committee accepted for the utility value for PFS off treatment both the EAG preferred value (0.71) and the company preferred value (0.76) – *vignette study + assumptions*<br>**TA380** (EQ-5D from Acaster et al. (58) (pre-progression no treatment) and van Agthoven et al. (59) (post-progression); EORTC QLQ-C30 from PANORAMA-1 to EQ-5D using Proskorovsky et al. (34) (pre-progression with treatment)) – *mapping + literature*<br>**TA552** (vignettes evaluated by members of the public using the TTO method; Hensen et al. (60) for post-transplant remission health state (vignette-based TTO study evaluated by members of the public)) – *vignette study + literature (vignette study)*<br>**TA523** (EQ-5D from Pan et al. (61) (relapse); Leunis et al. (62) (remission); Batty et al. (63) and Uyl-De Groot et al. (64) (treatment substates); mapping of EORTC QLQ-C30 from Grulke et al. (65) to EQ-5D using Crott and Briggs (66) for stem cell transplant substates) – *mapping + literature*<br>**TA640** (mapping of EORTC QLQ-C30 from Grulke et al. (65) to EQ-5D using Proskorovsky et al. (34); TA399/TA545 and Proskorovsky et al. (34) (relapse/progression); GvHD disutilities (Kurosawa et al. (9))) – *mapping + literature*<br>**TA667** (mapping of SF-36 from Burns et al. (67) to EQ-5D using Rowen et al. (32) (remission health state); multipliers for acute hospitalisation (true relapse health state), post-hospital discharge, and long-term complications were derived from a range of proxy conditions from the published literature) – *mapping + literature*<br>**TA748** (SF-36 from Statland et al. (68) to EQ-5D using the random effects GLS algorithm (model 3 in (68)); vignettes evaluated by members of the public using the TTO method) → the committee agreed that the utility increase from mexiletine is between the

Table 5: Appraisals in which EQ-5D is not available from a relevant study (n=66)

If EQ-5D is not available from relevant study, use utility values…	N (%)	Appraisals
		values generated by these 2 approaches – *assumptions/calculations*<br>**TA756** (manufacturer argued that the EQ-5D would not capture key symptoms of the condition and used utility values from a condition-specific preference-based measure (MF-8D from JAKARTA-2), this measure was previously developed and used in TA386; utility values for the AML and palliative care state from Pan et al. (61)) – *condition-specific preference-based measure* + *literature*<br>**TA873** (manufacturer considered EQ-5D to be insufficiently sensitive in patients with severe epilepsy and not able to capture the impact of small changes in seizure frequency → manufacturer used vignettes evaluated by members of the public using the TTO method and published literature (vignette study by Lo et al. (69) in which members of the public evaluated vignettes using the TTO method) – *vignette study* + *literature*

Appraisal identifiers highlighted in yellow indicate that some HSUVs were derived from a proxy condition

Table 6 gives an overview of the source of patient health state utility values in appraisals where evidence showed that EQ-5D was not appropriate.

Table 6: Source of patient health state utility values if evidence shows EQ-5D is not appropriate (n=7)

If evidence shows EQ-5D is not appropriate then use in order of preference...	N (%)	Appraisals
...other generic preference-based measure	-	**-**
...condition-specific preference-based measure	-	-
...vignettes	4 (3.6)	**HST12** (manufacturer did not include utility data (PedsQL, EQ-5D, CLN2-based QoL instrument) collected in the clinical studies (190-201/202) in the model because they were not available for all health states and for people receiving standard care → manufacturer used vignettes evaluated by clinical experts using EQ-5D)<br>**HST13** (EQ-5D and SF-36 from trial (APPROACH and APPROACH OLE) showed no statistically significant change from baseline and did not fully capture the effect on QoL → manufacturer used vignettes)<br>**HST23 (1)** and **HST23 (2)** (manufacturer did not use EQ-5D collected in the managed access agreement in the model due to lack of estimates for people in most severe health states → manufacturer used vignettes evaluated by clinical experts using EQ-5D)
...direct valuation of own health	-	-
...from the literature	*2 (1.8%)*	**HST16** (EQ-5D data from trial (ENVISION) did not show statistically significant difference between treatment arms and fewer attacks did not lead to improved HRQoL which was unexpected → manufacturer used utility vales from literature: EQ-5D values from relapsing-remitting multiple sclerosis as proxy for chronic symptoms from Hawton and Green (70), and EQ-5D values from a natural history study of people with AHP (EXPLORE) for acute attacks)<br>**TA804** (EQ-5D, SF-36, IBDQ, from trial (004) did not show difference in QoL between trial arms, SBS-QoL from trial (STEPS) did not show a statistically significant difference between trial arms → manufacturer used vignette study by Ballinger et al. (71) in which members of the public evaluated vignettes using the TTO method (in the vignette study, health states had 8 attributes, 3 associated with SBS-IF and home PS, and 5 described the 5 EQ-5D domains (72)))
...clinical expert input	*1 (0.9%)*	**TA755 (2)** (EQ-5D from trial (SUNFISH) available but not considered to be clinically plausible by clinical experts consulted by the manufacturer → manufacturer used estimates from clinical experts in TA588; disutilities due to disease complications sourced from the SUNFISH trial and Lloyd et al. (42))

Appraisal identifiers highlighted in yellow indicate that some HSUVs were derived from proxy conditions

S7 Carer health-related quality of life

Table 7: Appraisals in which EQ-5D is available for carer HRQoL (n=1)

EQ-5D is available…	**N (%)**	**Appraisals**
…from a relevant study	*1 (3.4%)*	**TA808** (EQ-5D from trials (study 1 and study 1504)

HRQoL = health-related quality of life

Table 8 gives an overview of appraisals in which EQ-5D data was not available from a relevant study.

Table 8: Appraisals in which EQ-5D is not available from a relevant study (n=28)

**If EQ-5D is not available from relevant study, use utility values…**	**N (%)**	**Appraisals**
…from the literature	6 (20.6)	**HST14 (1) and HST14 (2)** (carer HRQoL decrement was estimated as the difference between the mean value for carers in the manufacturers' Lipodystrophy Caregiver Burden Survey (EQ-5D) and the EQ-5D score for the general population)<br>**HST18 (1) and HST18 (2)** (EQ-5D from 73))<br>**HST25** (EQ-5D from manufacturers' observational study on caregiver health status comparing the burden on caregivers responsible for children with abnormal kidney function versus those responsible for children with normal kidney function)<br>**TA809** (EQ-5D from Thomas et al. (74))
…derived from statistical mapping	-	-
…vignettes	4 (13.8)	**HST28** (the manufacturer commissioned an online cross-sectional study to elicit carer HRQoL (EQ-5D recorded for 11 carers) but did not believe the results to be robust due to small sample size) → manufacturer used vignettes evaluated by the general public using the TTO method<br>**TA614** (vignettes evaluated by carers using VAS)<br>**TA615** (vignettes evaluated by carers using VAS)<br>**TA873** (vignettes evaluated by members of the public using TTO method)
…utility values from a 'proxy' condition	12 (41.4)	**HST9** (EQ-5D from Gani et al. (75)) → proxy condition: multiple sclerosis<br>**HST10** (value from TA217) → proxy condition: alzheimer's disease<br>**HST11** (EQ-5D from Al-Janabi et al. (76)) → proxy condition: meningitis<br>**HST13** (assumption informed by disutility values used in HST14 and Wittenberg and Prosser (77)) → proxy conditions: lipodystrophy and new musculoskeletal conditions<br>**HST16** (EQ-5D from Acaster et al. (78)) → proxy condition: multiple sclerosis<br>**HST17** (values and assumptions based on TA588 and TA534) → proxy conditions: spinal muscular atrophy and moderate to severe atopic dermatitis

Table 8: Appraisals in which EQ-5D is not available from a relevant study (n=28)

If EQ-5D is not available from relevant study, use utility values…	N (%)	Appraisals
		**HST19** (EQ-5D from Gani et al. (75) that informed original appraisal (HST2)) → proxy condition: multiple sclerosis<br>**HST20** (EQ-5D from Kuhlthau et al. (79) that was used in HST8) → proxy condition: children with activity limitations<br>**HST23 (2)** (EQ-5D from Landfeldt et al. (80) and HST3) → proxy condition: Duchenne muscular dystrophy<br>**HST26** (EQ-5D from Acaster et al. (78)) → proxy condition: multiple sclerosis<br>**HST29** (EQ-5D from Gani et al. (75), clinical expert input) → proxy condition: multiple sclerosis<br>**TA667** (EQ-5D from van Exel et al. (81)) →proxy condition: stroke
…calculations/ assumptions	*4 (13.9%)*	**TA588 (1) and TA588 (2)** (carer HRQoL decrement based on a range defined by the average utility from Spanish caregivers in López-Bastida et al. (41) and the EQ-5D score for the general population)<br>**TA755 (1) and TA755 (2)** (carer HRQoL decrement based on a range defined by the average utility from Spanish caregivers in López-Bastida et al. (41) and the EQ-5D score for the general population)
…a combination of sources	*2 (6.9%)*	**HST12** (EQ-5D for health state 6 from the literature (ICON study), expert input for health states 1 and 2, assumptions/calculations for remaining health status (assumption of a linear relationship between CLN2 clinical rating scale score and carer disutility, with the value for health state 6 from ICON study as mid-point of health states 3 to 9)) – *literature* + *expert input* + *calculations/assumptions*<br>**TA804** (carer utilities derived from a Delphi panel with clinical experts, EQ-5D from the manufacturer's carer quality of life survey) → results from the survey were grouped as per the Delphi estimates and then midpoints between the Delphi and grouped caregiver-specific estimates were taken – *expert input* + *carer survey* + *calculations/assumptions*

HRQoL = health-related quality of life

References

1. National Institute for Health and Care Excellence (NICE). NICE health technology evaluations: the manual. Manchester, UK: NICE; 2022 [last updated 14.07.2025]. Accessed July 28, 2025. https://www.nice.org.uk/process/pmg36/resources/nice-health-technology-evaluations-the-manual-pdf-72286779244741
2. Lampe C, Jain M, Olaye A, et al. Relationship Between Patient-Reported Outcomes and Clinical Outcomes in Patients With Morquio A Syndrome. *J Inborn Erros Metab Screen*. 2015;3. doi: 10.1177/2326409815576188
3. Pidala J, Kurland B, Chai X, et al. Patient-reported quality of life is associated with severity of chronic graft-versus-host disease as measured by NIH criteria: report on baseline data from the Chronic GVHD Consortium. *Blood*. 2011;117(17):4651-7. doi: 10.1182/blood-2010-11-319509
4. Wehler E, Storm M, Kowal S, et al. A Health State Utility Model Estimating the Impact of Ivosidenib on Quality of Life in Patients with Relapsed/Refractory Acute Myeloid Leukemia. *HemaSphere*. 2018;2(661). Accessed July 30, 2025. https://investor.agios.com/static-files/25de7161-9a10-4d8c-8a03-8bd5e7d0a5d3
5. Wild D, Walker M, Pettengell R, et al. PCN62 Utility elicitation in patients with follicular lymphoma. *Value Health*. 2006;9(6):A294. doi: 10.1016/S1098-3015(10)63491-2
6. Wild D. Utility values in Follicular Lymphoma - Oxford Outcomes [unpublished report by Oxford Outcomes prepared for Roche UK]. Oxford: Oxford Outcomes; 2005.
7. Swinburn P, Shingler S, Acaster S, et al. Health utilities in relation to treatment response and adverse events in relapsed/refractory Hodgkin lymphoma and systemic anaplastic large cell lymphoma. *Leuk Lymphoma*. 2015;56(6):1839-45. doi: 10.3109/10428194.2014.970542
8. Aristides M, Barlev A, Barber B, et al. Population preference values for health states in relapsed or refractory B-precursor acute lymphoblastic leukemia in the United Kingdom. *Health Qual Life Outcomes*. 2015;13:181. doi: 10.1186/s12955-015-0377-3
9. Kurosawa S, Yamaguchi H, Yamaguchi T, et al. Decision Analysis of Postremission Therapy in Cytogenetically Intermediate-Risk Acute Myeloid Leukemia: The Impact of FLT3 Internal Tandem Duplication, Nucleophosmin, and CCAAT/Enhancer Binding Protein Alpha. *Biol Blood Marrow Transplant*. 2016;22(6):1125-32. doi: 10.1016/j.bbmt.2016.03.015
10. Kelly MJ, Pauker SG, Parsons SK. Using nonrandomized studies to inform complex clinical decisions: the thorny issue of cranial radiation therapy for T-cell acute lymphoblastic leukemia. *Pediatr Blood Cancer*. 2015;62(5):790-7. doi: 10.1002/pbc.25451
11. Sung L, Buckstein R, Doyle JJ, et al. Treatment options for patients with acute myeloid leukemia with a matched sibling donor: a decision analysis. *Cancer*. 2003;97(3):592-600. doi: 10.1002/cncr.11098
12. Felder-Puig R, di Gallo A, Waldenmair M, et al. Health-related quality of life of pediatric patients receiving allogeneic stem cell or bone marrow transplantation: results of a longitudinal, multi-center study. *Bone Marrow Transplant*. 2006;38(2):119-26. doi: 10.1038/sj.bmt.1705417
13. van Agthoven M, Vellenga E, Fibbe WE, et al. Cost analysis and quality of life assessment comparing patients undergoing autologous peripheral blood stem cell transplantation or autologous bone marrow transplantation for refractory or relapsed non-Hodgkin's lymphoma or Hodgkin's disease. a prospective randomised trial. *Eur J Cancer*. 2001;37(14):1781-9. doi: 10.1016/s0959-8049(01)00198-8

14. Longworth L, Rowen D. Mapping to obtain EQ-5D utility values for use in NICE health technology assessments. *Value Health*. 2013;16(1):202-10. doi: 10.1016/j.jval.2012.10.010
15. Szabo SM, Levy AR, Davis C, et al. A multinational study of health state preference values associated with chronic myelogenous leukemia. *Value Health*. 2010;13(1):103-11. doi: 10.1111/j.1524-4733.2009.00573.x
16. Rombach SM, Smid BE, Bouwman MG, et al. Long term enzyme replacement therapy for Fabry disease: effectiveness on kidney, heart and brain. *Orphanet J Rare Dis*. 2013;8(47). doi: 10.1186/1750-1172-8-47
17. Lloyd AJ, Gallop K, MacCulloch A, et al. Estimating the Value of Treatment for Fabry Disease: A Discrete Choice Experiment. *Value Health*. 2016;19(7):A593-4. doi: 10.1016/j.jval.2016.09.1423
18. Younossi ZM, Boparai N, McCormick M, et al. Assessment of utilities and health-related quality of life in patients with chronic liver disease. *Am J Gastroenterol*. 2001;96(2):579-83. doi: 10.1111/j.1572-0241.2001.03537.x
19. Wright M, Grieve R, Roberts J, et al. Health benefits of antiviral therapy for mild chronic hepatitis C: randomised controlled trial and economic evaluation. *Health Technol Assess*. 2006;10(21):1-113, iii. doi: 10.3310/hta10210
20. Nordenfelt P, Dawson S, Wahlgren CF, et al. Quantifying the burden of disease and perceived health state in patients with hereditary angioedema in Sweden. *Allergy Asthma Proc*. 2014;35(2):185-90. doi: 10.2500/aap.2014.35.3738
21. Jørgensen T, Franchi A, Lelli F, et al. Preferences for Route of Administration, Frequency and Location - A Time-Trade-Off Study in the Italian General Population. *Value Health*. 2017;20(9):A637. doi: 10.1016/j.jval.2017.08.1444
22. Cooper JT, Lloyd A, Sanchez JJG, et al. Health related quality of life utility weights for economic evaluation through different stages of chronic kidney disease: a systematic literature review. *Health Qual Life Outcomes*. 2020;18(1):310. doi: 10.1186/s12955-020-01559-x
23. Sehn LH, Martelli M, Trněný M, et al. A randomized, open-label, Phase III study of obinutuzumab or rituximab plus CHOP in patients with previously untreated diffuse large B-Cell lymphoma: final analysis of GOYA. *J Hematol Oncol*. 2020;13(1). doi:
24. Lin JK, Muffly LS, Spinner MA, et al. Cost Effectiveness of Chimeric Antigen Receptor T-Cell Therapy in Multiply Relapsed or Refractory Adult Large B-Cell Lymphoma. *J Clin Oncol*. 2019;37(24):2105-19. doi: 10.1200/jco.18.02079
25. Landfeldt E, Lindberg C, Sejersen T. Improvements in health status and utility associated with ataluren for the treatment of nonsense mutation Duchenne muscular dystrophy. *Muscle Nerve*. 2020;61(3):363-8. doi: 10.1002/mus.26787
26. Brown MM, Brown GC, Sharma S, et al. Quality of life associated with visual loss: a time tradeoff utility analysis comparison with medical health states. *Ophthalmology*. 2003;110(6):1076-81. doi: 10.1016/s0161-6420(03)00254-9
27. Fordham BA, Kerr C, de Freitas HM, et al. Health state utility valuation in radioactive iodine-refractory differentiated thyroid cancer. *Patient Prefer Adherence*. 2015;9:1561-72. doi: 10.2147/ppa.S90425
28. Portwine C, Rae C, Davis J, et al. Health-Related Quality of Life in Survivors of High-Risk Neuroblastoma After Stem Cell Transplant: A National Population-Based Perspective. *Pediatr Blood Cancer*. 2016;63(9):1615-21. doi: 10.1002/pbc.26063
29. Brazier JE, Roberts J. The estimation of a preference-based measure of health from the SF-12. *Med Care*. 2004;42(9):851-9. doi: 10.1097/01.mlr.0000135827.18610.0d
30. Longworth L, Yang Y, Young T, et al. Use of generic and condition-specific measures of health-related quality of life in NICE decision-making: a systematic review, statistical modelling and survey. *Health Technol Assess*. 2014;18(9):1-224. doi: 10.3310/hta18090

31. Freemantle N, Wilson A, Fisher M. Mapping The St George's Respiratory Questionnaire To The Euroqol 5 Dimensions: A Study In Patients With Idiopathic Pulmonary Fibrosis. 2015;18(7):A503. Accessed July 29, 2025. https://www.valueinhealthjournal.com/article/S1098-3015(15)03507-X/fulltext
32. Rowen D, Brazier J, Roberts J. Mapping SF-36 onto the EQ-5D index: how reliable is the relationship? *Health Qual Life Outcomes*. 2009;7(1):27. doi: 10.1186/1477-7525-7-27
33. Guadagnolo BA, Punglia RS, Kuntz KM, et al. Cost-effectiveness analysis of computerized tomography in the routine follow-up of patients after primary treatment for Hodgkin's disease. *J Clin Oncol*. 2006;24(25):4116-22. doi: 10.1200/jco.2006.07.0409
34. Proskorovsky I, Lewis P, Williams CD, et al. Mapping EORTC QLQ-C30 and QLQ-MY20 to EQ-5D in patients with multiple myeloma. *Health Qual Life Outcomes*. 2014;12(35). doi: 10.1186/1477-7525-12-35
35. Gray AM, Rivero-Arias O, Clarke PM. Estimating the association between SF-12 responses and EQ-5D utility values by response mapping. *Med Decis Making*. 2006;26(1):18-29. doi: 10.1177/0272989x05284108
36. Matza LS, Sapra SJ, Dillon JF, et al. Health state utilities associated with attributes of treatments for hepatitis C. *Eur J Health Econ*. 2015;16(9):1005-18. doi: 10.1007/s10198-014-0649-6
37. Matza LS, Cong Z, Chung K, et al. Utilities associated with subcutaneous injections and intravenous infusions for treatment of patients with bone metastases. *Patient Prefer Adherence*. 2013;7:855-65. doi: 10.2147/ppa.S44947
38. Paracha N, Abdulla A, MacGilchrist KS. Systematic review of health state utility values in metastatic non-small cell lung cancer with a focus on previously treated patients. *Health Qual Life Outcomes*. 2018;16(1):179. doi: 10.1186/s12955-018-0994-8
39. Cherry MG, Greenhalgh J, Osipenko L, et al. The clinical effectiveness and cost-effectiveness of primary stroke prevention in children with sickle cell disease: a systematic review and economic evaluation. *Health Technol Assess*. 2012;16(43):1-129. doi: 10.3310/hta16430
40. Khan KA, Petrou S, Rivero-Arias O, et al. Mapping EQ-5D utility scores from the PedsQL™ generic core scales. *PharmacoEconomics*. 2014;32(7):693-706. doi: 10.1007/s40273-014-0153-y
41. López-Bastida J, Peña-Longobardo LM, Aranda-Reneo I, et al. Social/economic costs and health-related quality of life in patients with spinal muscular atrophy (SMA) in Spain. *Orphanet J Rare Dis*. 2017;12(1):141. doi: 10.1186/s13023-017-0695-0
42. Lloyd AJ, Thompson R, Gallop K, et al. Estimation Of The Quality Of Life Benefits Associated With Treatment For Spinal Muscular Atrophy. *Clinicoecon Outcomes Res*. 2019;11:615-22. doi: 10.2147/ceor.S214084
43. Stewart M, Mundayat R, Alvir J, et al. Clinical Characteristics and Health State Utilities in Patients With Transthyretin Familial Amyloid Polyneuropathy in Brazil. *Value Health*. 2017;20(5). Accessed July 29, 2025. https://www.ispor.org/heor-resources/presentations-database/presentation/ispor-22nd-annual-international-meeting/clinical-characteristics-and-health-state-utilities-in-patients-with-transthyretin-familial-amyloid-polyneuropathy-in-brazil
44. Rentz AM, Kowalski JW, Walt JG, et al. Development of a preference-based index from the National Eye Institute Visual Function Questionnaire-25. *JAMA Ophthalmol*. 2014;132(3):310-8. doi: 10.1001/jamaophthalmol.2013.7639
45. Thompson R, Vaidya S, Teynor M. The Utility of Different Approaches to Developing Health Utilities Data in Childhood Rare Diseases - A Case Study in Spinal Muscular Atrophy (SMA). *Value Health*. 2017;20(9):A725-6. doi: 10.1016/j.jval.2017.08.1962

46. Institute for Clinical and Economic Review (ICER). Spinraza® and Zolgensma® for Spinal Muscular Atrophy: Effectiveness and Value. April 3, 2019. Accessed July 30, 2025. https://icer.org/wp-content/uploads/2020/10/ICER_SMA_Final_Evidence_Report_110220.pdf
47. Kamath BM, Chen Z, Romero R, et al. Quality of Life and Its Determinants in a Multicenter Cohort of Children with Alagille Syndrome. *J Pediatr*. 2015;167(2):390-6.e3. doi: 10.1016/j.jpeds.2015.04.077
48. Arseneau KO, Sultan S, Provenzale DT, et al. Do patient preferences influence decisions on treatment for patients with steroid-refractory ulcerative colitis? *Clin Gastroenterol Hepatol*. 2006;4(9):1135-42. doi: 10.1016/j.cgh.2006.05.003
49. Al-Uzri A, Matheson M, Gipson DS, et al. The impact of short stature on health-related quality of life in children with chronic kidney disease. *J Pediatr*. 2013;163(3):736-41.e1. doi: 10.1016/j.jpeds.2013.03.016
50. Kini SP, DeLong LK, Veledar E, et al. The impact of pruritus on quality of life: the skin equivalent of pain. *Arch Dermatol*. 2011;147(10):1153-6. doi: 10.1001/archdermatol.2011.178
51. Parmar A, Vandriel SM, Ng VL. Health-related quality of life after pediatric liver transplantation: A systematic review. *Liver Transpl*. 2017;23(3):361-74. doi: 10.1002/lt.24696
52. Riazi A, Shakoor S, Dundas I, et al. Health-related quality of life in a clinical sample of obese children and adolescents. *Health Qual Life Outcomes*. 2010;8(1):134. doi: 10.1186/1477-7525-8-134
53. Alsumali A, Eguale T, Bairdain S, et al. Cost-Effectiveness Analysis of Bariatric Surgery for Morbid Obesity. *Obes Surg*. 2018;28(8):2203-14. doi: 10.1007/s11695-017-3100-0
54. Søltoft F, Hammer M, Kragh N. The association of body mass index and health-related quality of life in the general population: data from the 2003 Health Survey of England. *Qual Life Res*. 2009;18(10):1293-9. doi: 10.1007/s11136-009-9541-8
55. Sullivan PW, Slejko JF, Sculpher MJ, et al. Catalogue of EQ-5D scores for the United Kingdom. *Med Decis Making*. 2011;31(6):800-4. doi: 10.1177/0272989x11401031
56. Anyanwu AC, McGuire A, Rogers CA, et al. Assessment of quality of life in lung transplantation using a simple generic tool. *Thorax*. 2001;56(3):218-22. doi: 10.1136/thorax.56.3.218
57. Bradley J, Blume S, Balp M, Elborn S. Incidence and resource utilisation of pulmonary exacerbations in patients with cystic fibrosis in the UK. Presented at European Respiratory Society (ERS) Annual Congress, 2010 18-22 Sep;Barcelona, Spain.
58. Acaster S, Gaugris S, Velikova G, et al. Impact of the treatment-free interval on health-related quality of life in patients with multiple myeloma: a UK cross-sectional survey. *Support Care Cancer*. 2013;21(2):599-607. doi: 10.1007/s00520-012-1548-y
59. van Agthoven M, Segeren CM, Buijt I, et al. A cost-utility analysis comparing intensive chemotherapy alone to intensive chemotherapy followed by myeloablative chemotherapy with autologous stem-cell rescue in newly diagnosed patients with stage II/III multiple myeloma; a prospective randomised phase III study. *Eur J Cancer*. 2004;40(8):1159-69. doi: 10.1016/j.ejca.2004.01.019
60. Hensen M, Joshi N, Xu W, et al. Assessment of utility values for treatment-related health states of acute myeloid leukemia in the United Kingdom. *Value Health*. 2017;20:A115.
61. Pan F, Peng S, Fleurence R, et al. Economic analysis of decitabine versus best supportive care in the treatment of intermediate- and high-risk myelodysplastic syndromes from a US payer perspective. *Clin Ther*. 2010;32(14):2444-56. doi: 10.1016/j.clinthera.2010.12.003

62. Leunis A, Redekop WK, Uyl-de Groot CA, et al. Impaired health-related quality of life in acute myeloid leukemia survivors: a single-center study. *Eur J Haematol*. 2014;93(3):198-206. doi: 10.1111/ejh.12324
63. Batty N, Wiles S, Kabalan M, et al. Decitabine is more cost effective than standard conventional induction therapy in elderly acute myeloid leukemia patients. *Blood*. 2013;122(21):2699. doi: 10.1182/blood.V122.21.2699.2699
64. Uyl-De Groot C, Lowenberg B, Vellenga E, et al. Cost-effectiveness and quality-of-life assessment of GM-CSF as an adjunct to intensive remission induction chemotherapy in elderly patients with acute myeloid leukaemia. *Br J Haematol*. 1998;100(4):629-36. doi: 10.1046/j.1365-2141.1998.00635.x
65. Grulke N, Albani C, Bailer H. Quality of life in patients before and after haematopoietic stem cell transplantation measured with the European Organization for Research and Treatment of Cancer (EORTC) Quality of Life Core Questionnaire QLQ-C30. *Bone Marrow Transplant*. 2012;47(4):473-82. doi: 10.1038/bmt.2011.107
66. Crott R, Briggs A. Mapping the QLQ-C30 quality of life cancer questionnaire to EQ-5D patient preferences. *Eur J Health Econ*. 2010;11(4):427-34. doi: 10.1007/s10198-010-0233-7
67. Burns D, Lee D, Vesely S, et al. Patient health-related quality of life associated with remission of aTTP. A regression analysis using non-randomised observational data from the Oklahoma TTP registry. 2018;21(S3):S469. Accessed July 29, 2025. https://www.valueinhealthjournal.com/article/S1098-3015(18)36068-6/fulltext
68. Statland JM, Bundy BN, Wang Y, et al. Mexiletine for symptoms and signs of myotonia in nondystrophic myotonia: a randomized controlled trial. *JAMA*. 2012;308(13):1357-65. doi: 10.1001/jama.2012.12607
69. Lo SH, Lloyd A, Marshall J, et al. Patient and Caregiver Health State Utilities in Lennox-Gastaut Syndrome and Dravet Syndrome. *Clin Ther*. 2021;43(11):1861-76.e16. doi: 10.1016/j.clinthera.2021.09.017
70. Hawton A, Green C. Health Utilities for Multiple Sclerosis. *Value Health*. 2016;19(4):460-8. doi: 10.1016/j.jval.2016.01.002
71. Ballinger R, Macey J, Lloyd A, et al. Measurement of Utilities Associated with Parenteral Support Requirement in Patients with Short Bowel Syndrome and Intestinal Failure. *Clin Ther*. 2018;40(11):1878-93.e1. doi: 10.1016/j.clinthera.2018.09.009
72. Kennedy C, Robertson C, Cruickshank M, et al. Teduglutide for treating short bowel syndrome [ID3937]. Aberdeen HTA Group. November 10, 2021. Accessed March 17, 2025. https://www.nice.org.uk/guidance/ta804/documents/committee-papers
73. Pang F, Shapovalov Y, Howie K, Wilds A, Calcagni C, Walz M. Caregiver-reported impact on quality of life and disease burden in patients diagnosed with metachromatic leukodystrophy: Results of an online survey and a qualitative interview (Poster). 16th Annual WORLD Symposium. Orlando, Florida, USA. 2020.
74. Thomas GP, Saunders CL, Roland MO, et al. Informal carers' health-related quality of life and patient experience in primary care: evidence from 195,364 carers in England responding to a national survey. *BMC Fam Pract*. 2015;16. doi: 10.1186/s12875-015-0277-y
75. Gani R, Giovannoni G, Bates D, et al. Cost-effectiveness analyses of natalizumab (Tysabri) compared with other disease-modifying therapies for people with highly active relapsing-remitting multiple sclerosis in the UK. *PharmacoEconomics*. 2008;26(7):617-27. doi: 10.2165/00019053-200826070-00008
76. Al-Janabi H, Van Exel J, Brouwer W, et al. Measuring Health Spillovers for Economic Evaluation: A Case Study in Meningitis. *Health Econ*. 2016;25(12):1529-44. doi: 10.1002/hec.3259

77. Wittenberg E, Prosser LA. Disutility of illness for caregivers and families: a systematic review of the literature. *PharmacoEconomics*. 2013;31(6):489-500. doi: 10.1007/s40273-013-0040-y
78. Acaster S, Perard R, Chauhan D, et al. A forgotten aspect of the NICE reference case: an observational study of the health related quality of life impact on caregivers of people with multiple sclerosis. *BMC Health Serv Res*. 2013;13(1):346. doi: 10.1186/1472-6963-13-346
79. Kuhlthau K, Kahn R, Hill KS, et al. The well-being of parental caregivers of children with activity limitations. *Matern Child Health J*. 2010;14(2):155-63. doi: 10.1007/s10995-008-0434-1
80. Landfeldt E, Lindgren P, Bell CF, et al. Quantifying the burden of caregiving in Duchenne muscular dystrophy. *J Neurol*. 2016;263(5):906-15. doi: 10.1007/s00415-016-8080-9
81. van Exel NJ, Koopmanschap MA, van den Berg B, et al. Burden of informal caregiving for stroke patients. Identification of caregivers at risk of adverse health effects. *Cerebrovasc Dis*. 2005;19(1):11-7. doi: 10.1159/000081906
